# Plant Growth Promoting and Biocontrol Activity of *Streptomyces* spp. as Endophytes

**DOI:** 10.3390/ijms19040952

**Published:** 2018-03-22

**Authors:** Sai Shiva Krishna Prasad Vurukonda, Davide Giovanardi, Emilio Stefani

**Affiliations:** Department of Life Sciences, University of Modena and Reggio Emilia, via Amendola 2, 42122 Reggio Emilia, Italy; davide.giovanardi@unimore.it

**Keywords:** actinobacteria, streptomycetes, plant growth promoting rhizobacteria, microbe–microbe interactions, microbial biocontrol agents

## Abstract

There has been many recent studies on the use of microbial antagonists to control diseases incited by soilborne and airborne plant pathogenic bacteria and fungi, in an attempt to replace existing methods of chemical control and avoid extensive use of fungicides, which often lead to resistance in plant pathogens. In agriculture, plant growth-promoting and biocontrol microorganisms have emerged as safe alternatives to chemical pesticides. *Streptomyces* spp. and their metabolites may have great potential as excellent agents for controlling various fungal and bacterial phytopathogens. Streptomycetes belong to the rhizosoil microbial communities and are efficient colonizers of plant tissues, from roots to the aerial parts. They are active producers of antibiotics and volatile organic compounds, both in soil and *in planta*, and this feature is helpful for identifying active antagonists of plant pathogens and can be used in several cropping systems as biocontrol agents. Additionally, their ability to promote plant growth has been demonstrated in a number of crops, thus inspiring the wide application of streptomycetes as biofertilizers to increase plant productivity. The present review highlights *Streptomyces* spp.-mediated functional traits, such as enhancement of plant growth and biocontrol of phytopathogens.

## 1. Introduction

Plants are extensively colonized by a range of beneficial microorganisms and acquire a variety of plant–microbe interactions. Some of these interactions are beneficial, whereas some are detrimental to the plant. The microorganisms grow on plants as a resource of nutrients or habitat niche. In one such symbiotic interaction, the roots of many plants are infected by specific fungi (mycorrhizal association), rhizobia, and actinobacteria (particularly streptomycetes) that help the plant to acquire nutrients from the soil [1,2].

Currently, microbial endophytic communities are the focus of several studies aimed at unraveling and clarifying their role as plant growth promoters and their involvement in plant health. Several different bacterial species have been identified colonizing plant tissues and vessels, from the root system up to the stem, leaves, and other plant organs. Most of them are described as producers of metabolites positively interfering with plant life, for example, by enhancing nutrient acquisition or by stimulating plant defense mechanisms towards pathogens [3]. Rhizobacteria and mycorrhizal fungi are among the microorganisms that have proved to be of highest efficacy in promoting plant growth and, therefore, crop productivity. Rhizosphere bacteria are able to enhance nutrient uptake from the rhizosoil by the plants that they colonize. For this reason, they might be considered efficient biofertilizers. In most cases, such growth-promoting rhizosphere bacteria belong to the following species: *Alcaligenes*, *Arthrobacter*, *Azospirillum*, *Azotobacter*, *Bacillus*, *Burkholderia*, *Enterobacter*, *Klebsiella*, *Pseudomonas*, and *Serratia* [4,5]. *Streptomyces* spp. also belongs to the rhizospheric microbial communities, but only very recently their ability to act as plant growth promoters has been emphasized [6]. Rhizobacteria are also frequently found endophytically in roots and other plant parts, showing their ability to colonize their hosts. In such cases, their plant-stimulating activity does not cease, but can continue in the colonized plant tissues [7,8]. Mycorrhizal associations (ecto- and endomycorrhizae) are also pivotally important in ensuring plant growth and biomass through increasing nutrient and water uptake and enhancing plant resistance to abiotic and biotic stresses [9].

Mycorrhiza and rhizobia are, therefore, natural miniature fertilizer factories, an economical and safe source of plant nutrients compared to synthetic chemical fertilizers, which substantially contribute to environmental pollution. These microorganisms can increase agricultural production and improve soil fertility and, therefore, have great potential as a supplementary, renewable, and environmentally friendly source of plant nutrients.

*Streptomyces* spp. include many saprophytes, some of them becoming beneficial plant endosymbionts, but also include a few plant pathogens. The filamentous and sporulating nature of *Streptomyces* allows them to survive during unfavorable environmental conditions. Therefore, they appear to compete more efficiently against many other microorganisms present in the rhizosoil. Streptomycetes produce various lytic enzymes during their metabolic processes. Such enzymes are able to degrade insoluble organic polymers, such as chitin and cellulose, breaking them to substituent sugars for binding and uptake by multiple ABC transporters [10,11,12,13].

Plant growth promotion and productivity stimulated by microbial endophytic communities are often associated with increased plant health, achieved through direct and/or plant-mediated control of plant pests and pathogens. A few studies reported that root-associated microbes, particularly mycorrhizae and/or rhizobacteria, might influence and change plant physiology such that the aboveground parts are less prone to attack by phytophagous insects [14]. Plant defense is then achieved by priming for enhanced expression of sequences regulated by the production of jasmonic acid, ethylene, or salicylic acid. In other cases, beneficial microbes, such as root-colonizing pseudomonads, may directly act against plant-feeding insects by producing volatile organic compounds (VOCs) that have insecticidal properties [15]. In various studies, most of the antagonistic relationships between beneficial microbes and pathogens have been successful in explaining efficient biocontrol activity against many fungal diseases [16]. In a number of studies, researchers have found that endophytic microorganisms may have a symbiotic association with their host plants. According to Benhamou et al. [17], the endophytic *Bacillus pumilus* efficiently protected pea plants from *Fusarium oxysporum* f. sp. *pisi*, the causal agent of *Fusarium* root rot. Similarly, Varma et al. [18] demonstrated the growth-promoting activity in various plants elicited by the endophytic fungus *Piriformospora indica*. These endophytic microorganisms provide real advantages to the host plants, for example, by enhancing the physiological activity of the plant or facilitating the uptake of nutrients from the soil. Thus, they may serve as biocontrol agents or plant growth promoters [19]. Among other microorganisms, a variety of actinomycetes inhabits a wide range of plants as endophytes [20,21,22,23,24,25]; therefore, such actinobacteria may have both the potential to serve as effective biocontrol agents and to be considered as efficient plant growth promoters [26,27,28]. The genus *Streptomyces* has been extensively studied and used for biocontrol of soilborne fungal pathogens because of its intense antagonistic activity through the production of various antifungal metabolites [29,30,31].

## 2. *Streptomyces* spp. as Endophytes

Streptomycetes are Gram-positive bacteria belonging to the order *Actinomycetales* and the family *Streptomycetaceae*; roughly, streptomycetes are represented by more than 570 different species [32]. Streptomycetes are aerobic and filamentous bacteria able to produce vegetative hyphae that eventually form a complex mycelium and are able to grow and colonize different substrates. They are spore-forming bacteria and their spores may aid the dispersion and dissemination of the microorganism [33]. The genus *Streptomyces* includes ten plant pathogenic species, most of which are causal agents of the common scab of potatoes [34]. In nature, streptomycetes have a quite widespread distribution and are found in soils of very different structure and chemistry, in surface waters, and in plants as rhizosphere colonizers or true endophytes. As endophytic microorganisms, they colonize the internal part of plants, mainly the root system and the xylem tissues of the stem, causing no apparent change to their host’s morphology and physiology [35,36]. In different natural environments, they often play a major role in nutrient cycling. They may also have a strong influence in the population structure of environmental microbial communities due to their ability to produce a large set of secondary metabolites, many of which are of clinical and biotechnological importance [37,38].

From the medical point of view, *Streptomyces* is the largest antibiotic-producing genus against clinical microorganisms (fungi and bacteria) and parasites. They also produce other clinically important bioactive compounds such as immunosuppressants [39]. Only very recently streptomycetes has been considered as a prospective biocontrol agent in agriculture. Indeed, their ability to produce antibiotics may be used to control plant pathogenic bacteria and fungi [40]. Interference competition, an important strategy in interspecific interactions, is the production of growth inhibitory secondary metabolites (for example, antibiotics, toxins, biosurfactants, volatiles, and others) that can suppress or kill microbial opponents [41,42]. This feature is particularly present in streptomycetes, thus suggesting their use in excluding plant pathogens from their crop plants.

Interestingly, in a few cases, their interactions with plants may lead to suppression of the innate plant responses to phytopathogens. Therefore, it is of great importance to choose and characterize single *Streptomyces* strains for possible use as microbial antagonists. This is conveniently done through extensive in vitro and in planta studies on the roles of their antibiotics and possible production of VOCs [43]. One of the most common metabolites in streptomycetes communities is geosmin, a bicyclic alcohol derivative of decalin that confers the typical “earthy” flavor to the substrates they colonize [44]. Geosmin may be regarded as a volatile organic compound of microbial origin to which the human nose is extremely sensitive [45]. Although geosmin has no known antibiotic activity and its adaptive significance is not yet known, this metabolite might have an important role in the biology of streptomycetes [46]; indeed, it is a well-conserved trait and the gene responsible is highly conserved among *Streptomyces* spp. [47]. Geosmin enables bacteria to adapt to various environments, such as microbial communities or the host, ultimately influencing bacterial competition and cooperation [48]. It also has the ability to induce selective growth of geosmin-utilizing bacteria [49].

Microbial endophytes that efficiently and stably colonize different plant tissues, from roots to all aerial parts, have been long known, although their pivotal importance in agriculture has become evident only in recent decades. The main roles of endophytic microorganisms were discussed around 20 years ago, when several authors focused on symbiotic microorganisms and their possible plant–microbe interactions from a systematic, ecological, and physiological point of view [50,51,52,53]. Later discovery of the metabolic potential of such endophytes *in planta*, their ability to efficiently compete with other endophytes (included plant pathogens), and their role in stimulating the expression or overexpression of plant genomic sequences involved in tolerance/resistance to plant stresses (abiotic and biotic) indicated that selected endophytes may be considered as very promising agents to control plant pests and diseases.

**Figure 1:**
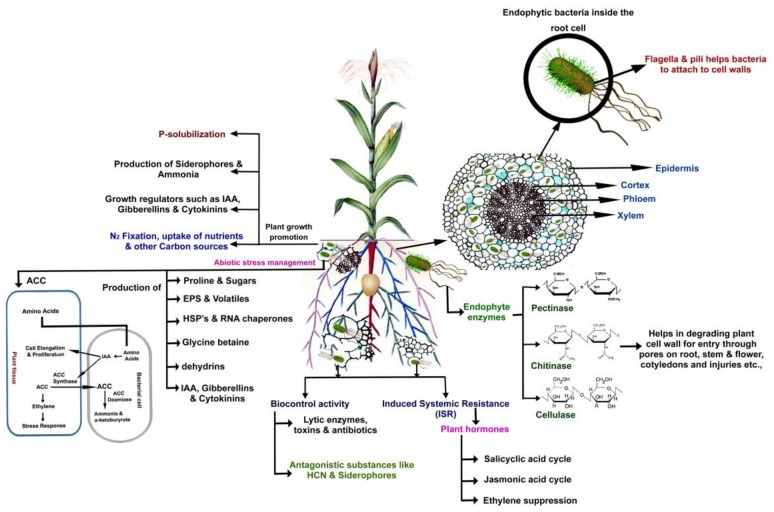
Representation of possible plant–microbe interactions favouring plant growth and/or biocontrol of phytopathogens by streptomycetes as rhizosphere competent microorganisms and/or endophytes (adapted from [57]).

Actinobacteria, and streptomycetes in particular, are known to constitute a large part of the rhizosoil microbiota. They may live saprophytically and endophytically in both natural and agricultural environments where they may colonize the rhizosphere and different morphological parts of plant roots [54]. Therefore, considering their plant growth-promoting activity, streptomycetes represent an excellent alternative for improving nutrient availability to crop plants and promoting innovation and sustainability in agricultural systems [55]. Plant growth-promoting streptomycetes (PGPS) stimulate and enhance several direct and indirect biosynthetic pathways in plants, for example, inorganic phosphate solubilisation, biosynthesis of chelating compounds, phytohormones production, inhibition of plant pathogens, and alleviation of various abiotic stresses (Figure 1) [56].

The isolation of actinomycetes in pure culture is an important step for screening the production of bioactive compounds. The most studied actinomycetes are species from the genus *Frankia*, a nitrogen-fixing bacterium of non-leguminous plants [58], and a few species of the genus *Streptomyces* that are phytopathogens [59]. Mundt and Hinckle [60] were able to isolate different species of *Streptomyces* and *Nocardia* from 27 different plant species, finding these actinobacteria present as endophytes in different plant tissues such as seeds and ovules. Sardi et al. [20] isolated and observed, through direct microscope examination, endophytic actinomycetes from the roots of 28 plant species from Northwestern Italy, finding actinomycetes belonging to the genus *Streptomyces* and other common genera, namely *Streptoverticillium*, *Nocardia*, *Micromonospora*, and *Streptosporangium*.

## 3. *Streptomyces* spp. as Plant Growth Promoters and Improvement of Plant Nutrition

Actinobacteria may have, in general, a positive role in plant mineral nutrition. This is correlated to both nitrogen fixation and metal mobilizing ability involving mineral nutrients such as Fe, Zn, and Se. Nonetheless, metagenomic analyses have not proven that streptomycetes are involved in such beneficial processes [61]. Metagenomic analyses of bacterial microbiota in plants have shown that the phylogenetic and taxonomic composition of such microbial communities is limited to few bacterial phyla, including actinobacteria.

More recently, Viaene et al. [7] highlighted the contribution of streptomycetes to plant growth and health. The plant has an important role in shaping its root microbiome through root exudate composition (chemotaxis) and nutritional interactions [62,63,64]. Plant root exudates are a source of metabolic signals (such as flavonoids, strigolactones, and terpenoids) that have the ability to shape the microbial communities in the rhizosphere. The signals that attract streptomycetes into the rhizosphere are still unknown. From the rhizosphere, streptomycetes are able to enter roots and colonize root tissues and vessels from where they can be isolated [24] and purified to identify them and describe their physiology and their microbe–microbe interactions.

Actinobacteria, such as *Streptomyces* spp., influence soil fertility through the involvement of many components and serve as nutrient enhancers. Besides producing siderophores and solubilizing phosphate, they are known to produce various enzymes—including amylase, chitinase, cellulase, invertase, lipase, keratinase, peroxidase, pectinase, protease, phytase, and xylanase—which make the complex nutrients into simple mineral forms. This nutrient cycling capacity makes them ideal candidates for natural fertilizers [65]. The relationship between PGPS and their host plant and the biochemical processes involved deserve deeper investigation. This knowledge would allow manipulation of those interactions, particularly the biochemical mechanisms leading to a compatible relationship between the host plant and its endophytes. Most streptomycetes are free-living in the soil as saprophytes and are able to colonize the rhizosphere and rhizoplane of the host plant. For instance, some PGPS, initially known as soil-dwelling microorganisms, were found to efficiently colonize the inner tissues of selected host plants as endophytes, therefore proving their ability to fully or partly conduct their life cycle inside plant tissues [66]. Additionally, a wide variety of *Streptomyces* species may establish beneficial plant–microbe interactions [67,68,69]. Table 1 summarizes the plant growth-promoting activity of *Streptomyces* species—many of them not fully identified—that gain access to root tissues from the rhizosoil. These species thus acquire an endophytic status without causing any visible harm or symptoms in the host plant. Such streptomycetes, although not always identified at the species level, are reported to have marked plant growth-promoting activity in their host plants. These species are most likely present in the apoplast of different parts of the plant (that is, roots, stems, leaves, flowers, fruits, and seeds) [69]. Coombs and Franco [70] demonstrated the endophytic colonization of wheat embryos, endosperm, and emerging radicles by tagging *Streptomyces* spp. strain EN27 with green fluorescent protein.

The endophytic streptomycetes can also be a source of metabolites that promote or improve host plant fitness and growth, as well as reduce disease symptoms that are caused by plant pathogens or various environmental stresses [71].

### Plant Hormone Production by Streptomycetes 

Many scientific reports have explained the ability of endophytic actinobacteria to stimulate the secretion of plant growth hormones and enhance their growth-promoting activity. A study by Dochhil et al. [97] described the evidence of plant growth-promoting activity and a higher percentage of seed germination due to the synthesis of higher concentrations (71 g/mL and 197 g/mL) of the plant growth hormone indole acetic acid (IAA) by two *Streptomyces* spp. strains isolated from *Centella asiatica*. In field trials, increased growth promotion and yield of cucumber was achieved by the application of *Streptomyces spiralis* alone, or in combination with other microbial “activators” such as *Actinoplanes campanulatus* or *Micromonospora chalcea*. Such experiments highlight the role of multiple microbes (or a microbial consortium) in very productive crop systems [98,99].

In soil, most of the known actinomycetes belong to genus *Streptomyces* and have been used for various agricultural purposes, mainly due to their production of antifungal and antibacterial metabolites and a number of plant growth-promoting (PGP) traits [100,101]. Indeed, more than 60% of known compounds with antimicrobial or plant growth-promoting activity originate from this genus [102]. In agricultural environments, *Streptomyces* species are an important group of soil bacteria because of their ample capacity to produce PGP substances, secondary metabolites (such as antibiotics), and enzymes [77,103].

Indole-3-acetic acid (IAA) is a common plant hormone belonging to the class of auxins. It has an important role in plant growth and development since it induces cell elongation and division. Manulis et al. [104] studied the production of IAA and the pathways of its synthesis by various *Streptomyces* spp., including *Streptomyces violaceus*, *Streptomyces griseus*, *Streptomyces exfoliates*, *Streptomyces coelicolor*, and *Streptomyces lividans*. Reddy et al. [105] isolated *Streptomyces atrovirens* from groundnut roots. This bacteria has shown excellent growth-promoting activity not only on groundnut but also on a number of other crops. These results are particularly interesting since they show the ability of a single streptomycete to promote growth in multiple different plants. El-Sayed et al. [106] and El-Shanshoury [107] reported IAA production in plants stimulated by *Streptomyces* sp. in greenhouse experiments while El-Tarabily [78] was successful in comparing different *Streptomyces* spp. strains. In these experiments, remarkably efficient growth promotion was stimulated by *Streptomyces filipinensis* due to its production of IAA.

1-aminocyclopropane-1-carboxylate (ACC) is a derived amino acid that is required for the endogenous biosynthesis of ethylene in plants. Comparing different streptomycetes, El-Tarabily [78] noted that the increased growth promoted by *Streptomyces filipinensis*, when compared to *S. atrovirens*, was due to the production of both IAA and ACC, whereas *S. atrovirens* produced only ACC deaminase. Therefore, a single streptomycetes was shown to produce more than one plant hormone. These results are of great interest for the possible exploitation of streptomycetes as plant growth stimulants.

The endophytic colonization of streptomycetes connected with their influence on plant nutrition is poorly studied so far. However, nitrogen-fixing actinobacteria and their correlation with plant nutrition and productivity have been recently described [108]. Among such actinobacterial communities, a few *Streptomyces* spp. with nitrogen-fixing capacity have been identified. Siderophore production has also been described. In particular, isolates of *Streptomyces* spp. were able to produce and excrete an enterobactin, an iron-chelating compound characteristic of some *Enterobacteriaceae* [90,109].

## 4. Streptomycetes in Plant Protection against Biotic Stresses

Microbial biocontrol agents have the ability to perform antibiosis, parasitism, or competition with the pathogen for nutrients and space. They may also induce disease resistance in the host plant that they colonize, acting along different steps of the infection process. Therefore, protection of plants from biotic stresses may be the result of one or more microbe–microbe or plant–microbe interactions [110]. Actinomycetes, and particularly *Streptomyces* species, are well known for their production of a wide spectrum of antibiotics. These are often species specific and allow them to develop symbiotic interactions with plants by protecting them from various pathogens; at the same time, plant exudates promote *Streptomyces* growth [111]. In the last two decades, there has been an increasing interest in antibiosis by PGPB and such biocontrol mechanisms are now better understood [112]. Several metabolites with antibiotic nature produced by pseudomonads have been studied and characterized so far, e.g., the cyclic lipopeptide amphysin, 2,4-diacetylphloroglucinol (DAPG), oomycin A, the aromatic polyketide pyoluteorin, pyrrolnitrin, the antibacterial compound tropolone [113,114]. Other bacterial genera, such as *Bacillus*, *Streptomyces*, *Stenotrophomonas* spp., produce the macrolide oligomycin A, kanosamine, the linear aminopolyol zwittermicin A, and xanthobactin [115,116]. They also synthesize several enzymes that are able to disrupt fungal cell walls [39]. Early studies performed during the 1950s described the production by streptomycetes of a set of antibiotics suitable for controlling foliage diseases caused by phytopathogenic fungi [117,118]. Later, several other authors reported excellent biocontrol activity of some phytopathogenic soilborne fungi such as *Pythium* spp., *Fusarium* spp. [119], *Rhizoctonia solani* [120], and *Phytophthora* spp. [121] (Table 2). 

**Figure 2:**
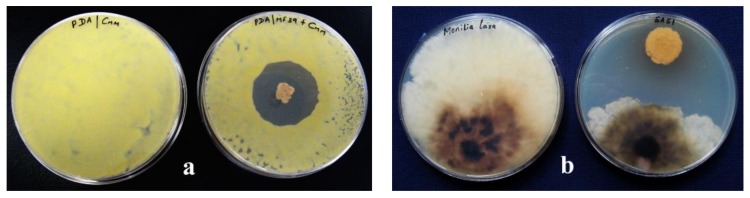
In vitro biocontrol activity of *Streptomyces* spp.: (**a**) antimicrobial activity against *Clavibacter michiganensis* subsp. *michiganensis*, the causal agent of the tomato bacterial canker and (**b**) antifungal activity against *Monilinia laxa*, the causal agent of the brown rot of stone fruits.

As shown in Table 2, streptomycetes are promising microbial biocontrol organisms that are able to antagonize and/or kill fungal and bacterial plant pathogens. Their biocontrol activity is often performed before the pathogens completely infect their respective host. Recently, these organisms have been the focus of different approaches toward the development of biocontrol strategies against soilborne pathogens [120,169]. For instance, by treating seeds with endophytic *Streptomyces* spp. and *Micromonospora* spp. prior to sowing, *Arabidopsis thaliana* was protected from infection by *Erwinia carotovora* and *F. oxysporum*. Streptomycetes were observed antagonizing pathogens by inducing the expression of defense pathways in the plant [170]. This observation implies that the microbial antagonists penetrated the seeds during their germination and colonized the seedlings. Bacon and Hinton [171] reported that varying levels of disease suppression in the field were positively correlated with similar results obtained from in vitro experiments. In other experiments, a significant pathogen inhibition in vitro was not always correlated with disease protection *in planta*. Growth inhibition of plant pathogens by endophytic bacteria indicates the presence of antagonistic activities between them, which may act directly (by mechanisms of antibiosis, competition, and lysis) or indirectly (by inducing plant defense or by growth-promoting substances) [31,172] (Figure 2a,b).

Production of chitinolytic enzymes and siderophores (iron-chelating compounds) is a known additional mode of action for fungal growth inhibition by endophytic actinobacteria. Endophytic actinobacteria can produce enzymes that degrade fungal cell walls, especially by the production of chitinases. Over 90% of chitinolytic microorganisms are actinomycetes. These have been extensively studied during the last two decades, starting in the mid-1990s [173]. The production of chitinases by actinomycetes and by streptomycetes in particular makes these organisms very promising microbial biocontrol agents. In *Streptomyces plicatus*, chitinases are encoded in a region of *chi65*, the expression of which is induced by *N*,*N*′-diacetylchitobiose and activated by allosamidin [174]. In fungi, chitinase is necessary for fungal development, such as hyphal growth and branching [175]. Several bacteria, and streptomycetes in particular, also produce a set of chitinases to obtain nutrients through degradation of environmental chitin, including the cell wall of soil fungi. Therefore, this ability may be exploited in the selection and exploitation of chitinolytic microbial agents for the biocontrol of phytopathogenic fungi [176,177]. Allosamidin is an important secondary metabolite of streptomycetes and was initially reported as a chitinase inhibitor [178]. Later, they further investigated the role of allosamidin in its producing *Streptomyces* and showed that allosamidin inhibits all family 18 chitinases, but can dramatically promote chitinase production and growth of its producer *Streptomyces* [174,179]. This appears particularly important for the bacterial growth in soil where chitin, mainly originating from insect cuticle and fungal cell walls, is a major nutrient source. Therefore, allosamidin is listed as a potent secondary metabolite with antifungal activity [180].

Although both crude and purified chitinases have great potential for cell wall lysis of fungal pathogens, in common agricultural systems the use of selected streptomycetes as microbial biocontrol agents targeting important phytopathogens appears to be a more effective strategy. This is due to the high cost of purified antimicrobial molecules, making them more suitable as pharmaceuticals against clinical and animal pathogens. The ability of siderophores to promote plant growth and enhance antagonism to phytopathogens has gained more significance [69,181,182]. El-Shatoury et al. [183] reported actinobacteria from *Achillea fragrantissima* that were capable of producing both chitinases and siderophores; they also showed remarkable inhibitory activity against phytopathogenic fungi. These reports were strongly supported and further explained by Gangwar et al. [184] studying actinobacteria from *Aloe vera*, *Mentha arvensis*, and *Ocimum sanctum*. The latter authors provided quantitative data for different types of siderophore compounds: the hydroxamate-type of siderophore ranged between 5.9 and 64.9 μg·mL^−1^ and the catechol-type of siderophore occurred in a range of 11.2–23.1 μg·mL^−1^. In another investigation, El-Tarabily et al. [185] applied endophytic *Streptomyces spiralis* together with *Actinoplanes campanulatus* and *Micromonospora chalcea* to cucumber seedlings. Since this group of microorganisms, applied as a microbial consortium, showed more effectiveness in the reduction of seedling damping off and root- and crown-rot diseases by *Pythium aphanidermatum* than the single actinobacterium, this study recommended their use as very effective biocontrol agents. Therefore, as for other PGPR, several streptomycetes produce siderophores to sequester iron in the rhizosphere, making iron unavailable to certain rhizoplane microorganisms, in particular to some phytopathogens. These pathogenic microorganisms are often unable to obtain essential quantities of iron for their growth because they do not produce siderophores, produce comparatively less siderophores than PGPR, and/or produce siderophores that have less affinity for iron than those of PGPR [186].

Igarashi studied the new bioactive compound 6-prenylindole produced by a *Streptomyces* spp. [187]. In the beginning, it was reported as a component of liverwort (*Hepaticae*) and it showed significant antifungal activity against *Alternaria brassicicola*. Interestingly, this molecule was isolated from both plants and microorganisms [187]. Similar reports by Zhang et al. [188] explained the inhibition of the phytopathogenic fungi *Colletotrichum orbiculare*, *Phytophthora capsici*, *Corynespora cassiicola*, and *Fusarium oxysporum* by a new prenylated compound and three known hybrid isoprenoids with IC_50_ in the range 30.55–89.62. Another study by Lu and Shen [189,190] reported inhibition of *Penicillium avellaneum* UC-4376 by naphthomycins A and K produced by *Streptomyces* spp. The synthesis of fistupyrone, a metabolite produced by *Streptomyces* spp. isolated from leaves of spring onion (*Allium fistulosum*), was found by Igarashi [187] to inhibit *Alternaria brassicicola*, the causal agent of the black leaf spot in *Brassica* plant. This study reported that fistupyrone was able to inhibit the fungal infection process by pre-treating the seedlings with such compound at a concentration of 100 ppm. In support of this statement, experiments by Igarashi et al. [191] evidenced that fistupyrone did not inhibit the growing hyphae but suppressed spore germination of fungi at 0.1 ppm concentration. 

*Streptomyces* spp. have the capacity to produce cellulolytic enzymes and various secondary metabolites, which directly act on herbivorous insects and show toxic activity on phytopathogens and/or insect pests [192,193]. A set of different molecules from *Streptomyces* spp. that act against insect pests have been found and characterized; these are, for instance, flavensomycin [194], antimycin A [195], piericidins [196], macrotetralides [197] and prasinons [198]. *Streptomyces avermitilis*, a common soil inhabitant, was shown to produce avermectins, molecules with potent activity against arthropods and nematodes [199]. These compounds derive from lactones and are macrocyclic in nature; they mainly act on the insect peripheral nervous system by targeting the γ-aminobutyric acid (GABA) receptors, leading to paralysis of the neuromuscular system [200]. Commercial insecticides based on avermectin mixtures are known as abamectin and they act on phytophagous arthropods directly by contact and ingestion. They are not systemic in plants, showing just a limited translaminar activity. Similar molecules produced by *Streptomyces* spp. are emamectin—particularly toxic to Lepidoptera, and milbemectin—and are specifically isolated from *S. hygroscopicus*.

## 5. Commercialization, Environmental Effects, and Biosafety of *Streptomyces* Products

Streptomycete producing antimicrobial secondary metabolites present an attractive alternative to chemical fertilizers, pesticides, and supplements, which may result in a significant increase in agricultural plant growth and pest and disease control [201]. While increasing our knowledge of the mechanisms triggered by actinomycetes for suppressing plant diseases, improving nutrient uptake by plants, and stimulating and/or increasing the production of phytohormones in planta, a great deal of research is being carried out worldwide for the development of correct formulations containing actinomycete inoculants as their active ingredients. Nevertheless, very few actinomycete-based products are currently commercialized. Although biocontrol with PGPR is an acceptable green approach, the proportion of registration of *Streptomyces* spp. as biocontrol agents for commercial availability is very low. Mycostop (Verdera Oy, Finland) is the only *Streptomyces*-based plant protection product registered in the EU; it is also registered in Canada and the USA. Actofit and Astur, based on *Streptomyces avermitilis*, are registered as insecticides in the Ukraine. Table 3 lists the microbial pesticides that are registered in particular countries worldwide. In most cases, metabolites produced by *Streptomyces* spp. are registered as active substances in plant-protection products, as shown in Table 4. 

Any formulation with an increased shelf life and a broad spectrum of actions, such as plant growth promotion and/or disease suppression under field conditions, could open the way for technological exploitation and marketing. Many reports suggest that commercial biocontrol agents are easy to deliver, induce plant growth and stress resistance and, eventually, increase plant biomass and yield. As very promising and rich sources of agro-active compounds and biocontrol tools, actinomycetes have gained increasing interest in several agricultural sectors [206,207]. In fact, in the last 30 years, about 60% of new insecticides and herbicides reported have originated from *Streptomyces* [206]; this is because three-quarters of all *Streptomyces* spp. are able to produce some class of antibiotics [208]. As a single example among many, we mention the production of polyoxin B and D by *Streptomyces cacaoi* var. *asoensis* as a new class of natural fungicides [209]. *Kasugamycin*, registered in several countries as a bactericidal and fungicidal agrochemical, was discovered in *Streptomyces kasugaensis* [210]. More recently, Siddique et al. [211] reported that avermectin B1b, a component of commercially available abamectin, was obtained as a fermentation product of *Streptomyces avermitilis*, which has frequently been used as an insecticidal agent. Very few *Streptomyces*-based commercial formulations are available in the worldwide market, compared with products based on *Streptomyces* metabolites; the former are mainly indicated for pest and disease control. Additionally, few products have been specifically commercialized for plant growth promotion, although significant research has been carried out on actinomycete production of growth-promoting substances [212]. One reason for this gap may reside in the difficulty of preparing a commercial product formulation with one or more streptomycetes as an active substance. Ideally, the industrial process used should not affect the bioproduct’s plant growth-promoting activity and/or antimicrobial characteristics for 18–24 months. 

In keeping with current quality and safety standards, ideal microbial biocontrol agents should be univocally identified as a taxonomical unit; be effective against target plant pathogens or pests; show no clinical or animal toxicity; should not persist in the agro-environment (included surface water), having a short growing period, a level-off, and a final lining to the background microorganisms; and should not transfer genetic material to other taxonomically related microorganisms. Therefore, the antagonistic potential, environmental fate, and behavioural features of a putative microbial biocontrol agent must be thoroughly addressed by the industry to allow its registration as a bio-pesticide and approval for use in plant protection. All this may hinder the transfer of an effective biocontrol agent from the research lab into a commercially available product. Indeed, the BCB Manual of Biocontrol Agents, 5th Edition, lists over 120 microorganisms with potential use in agriculture, 54 of which have been approved in the EU for use in plant protection; however, only a few are commercially available. A complex regulatory landscape must be navigated by applicants applying for authorization to release a biocontrol agent. This is particularly true if the biocontrol agent is not indigenous [213,214].

Environmental risks associated with the inoculation of streptomycetes in agricultural environments as organisms beneficial to plants are associated with the lack of available data concerning the use of genetically modified organisms and their impact on the natural microbial communities [215]. In addition, the release of not genetically modified microbials may pose a risk related to the possible horizontal transfer of entire or partial gene clusters; this might be particularly risky in the case of antibiotic resistance. This was initially reported by Egan et al. [216] and later confirmed by Egan et al. [217]. This risk might be minimized during the search and study of prospective microbial inoculants, which should be accurately tested and characterized prior to their registration for the lack of known antibiotic-resistant genes or gene clusters.

To ensure food security for an increasing worldwide human population, most agricultural systems presently depend on the use of chemical fertilizers and pesticides [218]. Industrially produced chemical fertilizers are rich in nitrogen, phosphorous, and potassium, the repeated use of which leads to pollution of the soil, air, and groundwater [219]. Given these known problems, beneficial agricultural microorganisms used as microbial inoculants will be an important focus in pursuing sustainable agriculture and the provision of safe food without depleting natural resources in the coming decades [220]. The application of these naturally occurring beneficial microorganisms to soil ecosystems improves the soil’s physical–chemical properties, fitness and stability, and microbial development along with promoting plant growth promotion and crop yield [221].

The microbial agents with the greatest agricultural prospects are rhizobacteria (as plant growth promoters), nitrogen-fixing cyanobacteria, mycorrhizal fungi, bacterial antagonists to plant pathogens, different biotic and abiotic stress-tolerance endophytes, and biodegrading bacteria [222,223]. Current registration and authorization procedures for microbe-based products to be used in agriculture as “fertilizers” are much less demanding; several microbe-based products or microbial consortia are, therefore, commercially available to farmers worldwide. Among them, a few contain streptomycetes and other *Actinomycetales*. Examples include Micosat F^®^ (CCS Aosta srl, Aosta, Italy), containing three different *Streptomyces* spp.; Forge SP^®^ (Blacksmith Bioscience, Spring, TX, USA), containing *Streptomyces nigrescens*; and Mykorrhyza soluble 30G (Glückspilze, Innsbruck, Austria), containing *Streptomyces griseus* and *S. lydicus*.

## 6. Formulations and Inoculation Methods

For any agro-pharma industry, the major challenge for the success or failure of a commercial product developed from an experimentally efficient biocontrol agent is its “formulation.” Formulations should include the novel microorganism(s) in a calibrated quantity as an active ingredient and a set of other inert ingredients (frequently not specified in detail by the manufacturer) to produce a commercial product suitable for use in field conditions. Commercial formulations should comply with local and national legislation on agrochemicals (specifically legislation on microbial inoculants, where applicable), as well as with growers’ requirements: repeated positive results, reasonable pricing, and easy handling. With regard to these considerations, microbial inoculants have a major problem specific to microorganisms: loss of viability during storage complicates the need for a long shelf life and stability over a range of −5 to 30 °C, which are typical growers’ storage conditions [224]. Commercial microbial formulations can be prepared and made available on the market in four types: powder, liquid suspension, granules, and slurry [225,226]. The physical formulation of *Streptomyces*-based products also indicates how these microbials should be inoculated in agricultural systems, prior or during cropping (Table 5). Different types of low-cost raw materials are used to prepare different types of commercial formulations: peat, perlite, charcoal, vermicompost, inorganic soil fractions, and many others [227].

Moreover, biocontrol agents may not show the same results in both in vitro and in vivo experiments; this is regarded as the crucial challenge in the industrial development of bio-inoculants. The efficacy of biocontrol agents, among them streptomycetes, is affected by soil organic matter, pH, nutrient levels, and moisture level. Variations in agri-environmental conditions tend to affect the results of biocontrol agents that perform well in vitro: an experimentally excellent biocontrol agent might fail in greenhouse or field experiments. Thus, environmental variables should always be taken into proper consideration when selecting an appropriate biocontrol agent for a precise location. Ideally, the most active and prospective microbes should be isolated from the same agricultural area [228].

Additionally, any physical formulation, method, or procedure of inoculation (for example, soil, seed, seedling, or vegetative part) should be thoroughly screened since each method may play an important role in obtaining satisfactory results during field experiments [228,229]. For instance, in inoculating soil with a biocontrol agent, microbes are mixed with soil or sowing furrows or are spread in the field by dripping systems [230]. Seed inoculation methods commonly involve soaking seeds in a suspension containing the selected biocontrol agent(s); alternatively, they are mixed with suitable wetting agents [228,229,230,231,232]. The inoculation of vegetative parts is done by spraying a suspension of the biocontrol agent to aerial parts of the plant or dipping the roots of seedlings into a microbial suspension prior to transplantation [230,232]. Each method of microbial application may contribute to achieving effective results in commercial agriculture (open field, nurseries, glasshouse production, etc.) [228,233]. (Table 5).

As described in previous chapters, most streptomycetes are soil inhabitants, commonly colonizing the rhizosphere and frequently showing the ability to enter plants, thus efficiently colonizing plant tissues as endophytes. Since pathogens necessarily require an endophytic state to initiate an infection, a successful endophytic biocontrol agent, such as a selected *Streptomyces* sp., should be able to rapidly move from the rhizosphere into the roots and/or other plant parts. Therefore, its antagonistic activity may be both preventive (competitive exclusion of plant pathogens in the rhizosphere and *in planta*) and curative (killing plant pathogens post-infection). From the industrial point of view, the selection and choice of prospective *Streptomyces* spp. as candidates for development and implementation of innovative and sustainable biopesticides should necessarily consider that goal.

Industrial exploitation of research results is not necessarily easy. Most papers published worldwide demonstrate the excellent biocontrol activity of streptomycetes during experiments in vitro or, when in planta, under strictly standardized conditions. Frequently, no field research results are presented to support the applicability of experimental data in commercial fields/greenhouses. Clearly this is a weak point, hindering the commercial development of successful biopesticides. Development is also hindered because the production of antimicrobial molecules by actinobacteria, and particularly by streptomycetes, is strictly dependent on the substrate where they grow [234] and on the natural microbial community around them.

## 7. Future Aspects and Challenges

*Streptomyces* spp. have great potential to become an essential constituent of modern agricultural practice as biofertilizers and biocontrol agents, with the capacity to dominate agrimarkets in coming decades. Actinomycetes, particularly abundant *Streptomyces* as filamentous spore-forming bacteria with superior biocontrol and nutrient-cycling activity, are among the most promising PGPR to increase overall soil health and boost agricultural productivity. Nevertheless, some unresolved problems need to be addressed in order to reproduce results from the controlled laboratory environment into large-scale field trials and commercial marketing. More focus is still needed to develop novel formulations that could increase the shelf life of streptomycetes, thus ensuring their long-term viability, their sporulation activity, and their efficacy as microbial-based agrochemicals. Additionally, the potential of streptomycetes to control post-harvest bacterial and fungal diseases of fruits and vegetables is totally unexplored. Further extensive studies on the complex *Streptomyces*–rhizosphere environment and the mechanisms of PGP action are needed. Shedding light on the symbiotic association of *Streptomyces* with other PGPR might lead to developing highly effective and efficient bioinoculants across different soil types and environmental conditions. The knowledge of various aspects such as interactions between rhizosphere PGPS and native microbiota and infection processes by endophytic PGPS are still not sufficiently explained, even though many reports have indicated that PGPS can promote plant growth by colonizing their host plants epiphytically and/or endophytically. Metagenomics and molecular biology studies, such as tagging green fluorescent protein (GFP) markers to microorganisms, will be necessary to understand the fate of PGPS microbial populations in plants, their endophytic distribution, and their pattern of colonization. Much more focus is still needed to design and implement industrial processes that are able to produce effective formulations with one or more microbial agents, using different additives, carriers, and with various methods of field inoculations.

## 8. Conclusions

Many studies have been conducted on actinomycetes, highlighting the ability of these microorganisms to promote plant growth and their additive/synergistic effects on plant growth and protection. As the above discussion makes clear, actinomycetes, and especially *Streptomyces* as helper bacteria, are truly prospective for use as plant coinoculants: this to improve plant–microbe symbiosis in a way that could lead to an increased sustainable production of agriculture products under diverse conditions. This promise is mainly based on the use of eco-friendly microorganisms that control pests and improve plant growth. The use of biofertilizers, biopesticides, or consortiums of plant beneficial microbes in correct formulations provides a potential solution for a more sustainable agricultural future. The studies mentioned in this review support the belief that designing new formulations with cooperative microbes might contribute to growth improvement and plant protection of several crops. However, these studies also highlight the importance of continuing research on this subject, especially focusing on actinomycetes, which up to now have been little used as inoculants to enhance agricultural production and ensuring food security, despite the excellent potential shown in a large number of scientific publications so far. 

## Figures and Tables

**Table 1:** List of streptomycetes isolated from plants or the rhizosphere showing plant growth-promoting (PGP) activity.

Species	Host Plant	PGP Traits/Observed Effects in Plants	Reference
*Streptomyces* sp.	Clover	Nutrient uptake	[72]
*Streptomyces* sp.	Rice, chickpea	Nutrient uptake and plant growth	[73,74]
*Streptomyces lydicus*	Pea	Nodulation	[75]
*Streptomyces* sp.	Mung bean	Enhanced plant growth	[68]
*Streptomyces* sp.	Soybean	Nutrient uptake and plant growth	[76]
*Streptomyces atrovirens*, *S. griseoviridis*, *S. lydicus*, *S. olivaceoviridis*, *S. rimosus*, *S. rochei*, *S. viridis*	Rhizosphere of different plants	Auxin/IAA production	[77,78,79,80,81]
*Streptomyces* sp.	-	Gibberellin biosynthesis	[82]
*Streptomyces igroscopicus*	-	ACC deaminase	[83]
*Streptomyces* sp.	*Terfezia leonis* Tul.	Siderophore production, IAA, and gibberellic acid production	[84]
*Streptomyces* sp.	Marine environments	Gibberellic acid, IAA, abscisic acid, kinetin, and benzyladenine	[85]
*Streptomyces aurantiogriseus*	Rice	IAA production	[86,87]
*Streptomyces* sp.	Soil	Synthesis of IAA and siderophore production	[88]
*Streptomyces* spp.	-	B-1,3-Glucanase, IAA, and HCN synthesis	[73,89]
*Streptomyces* sp.	-	Siderophore production	[90]
*Streptomyces rochei*, *S. carpinensis*, *S. thermolilacinus*	Wheat rhizosphere	Production of siderophore, IAA synthesis, and phosphate solubilization	[91]
*Streptomyces* sp.	Soil	Siderophore production, phosphate solubilization, and N_2_ fixation	[72]
*Streptomyces* spp.	*Alnus glutinosa*, *Casuarina glauca*, *Eleagnus angustifolia*	Production of zeatin, gibberellic acid, and IAA	[92]
*Streptomyces olivaceoviridis*, *S. rochei*	Wheat	Auxin, gibberellin, and cytokinin synthesis	[93]
*Streptomyces hygroscopicus*	Kidney beans	Formation of adventitious roots in hypocotyls	[94]
*Streptomyces* sp.	Rhododendron	Accelerated emergence and elongation of adventitious roots in tissue-cultured seedlings	[28]
*Streptomyces filipinensis*, *S. atrovirens*	Tomato	Plant growth promotion	[78]
*Streptomyces spiralis*	Cucumber	Plant growth promotion	[95]
*Streptomyces* spp.	Sorghum	Enhanced agronomic traits of sorghum	[96]
	Rice	Enhanced stover yield, grain yield, total dry matter, and root biomass	

Note: IAA: Indole-3-acetic acid; ACC: 1-amino cyclopropane-1-carboxylic acid; HCN: Hydrogen cyanide.

**Table 2:** Biocontrol activity of several *Streptomyces* spp. against different fungi.

Species/Strain	Plant	Disease	Target Pathogens	References
*Streptomyces viridodiasticus*	Lettuce	Basal drop disease	*Sclerotinia minor*	[122]
*S. violaceusniger* G10	Banana	Wilt	*Fusarium oxysporum* f. sp. *cubense* race 4	[123]
*Streptomyces* sp. KH-614	Rice	Blast	*Pyricularia oryzae*	[124]
*Streptomyces* sp. AP77	Porphyra	Red rot	*Pythium porphyrae*	[125]
*Streptomyces* sp. S30	Tomato	Damping off	*Rhizoctonia solani*	[126]
*S. halstedii*	Red pepper	Blight	*Phytophthora capsica*	[127]
*Streptomyces* spp. 47W08, 47W10	Pepper	Blight	*Phytophthora capsica*	[128]
*S. violaceusniger* XL-2	Many	Wood rot	*Phanerochaete chrysosporium*, *Postia placenta*, *Coriolus versicolor*, *Gloeophyllum trabeum*	[129]
*S. ambofaciens* S2	Red chili fruits	Anthracnose	*Colletotrichum gloeosporioides*	[130]
*Streptomyces* spp.	Sugar beet	Damping off	*Sclerotium rolfsii*	[131]
*S. hygroscopicus*	Many	Anthracnose and leaf blight	*Colletotrichum gloeosporioides* and *Sclerotium rolfsii*	[132]
*Streptomyces* spp.	Sunflower	Head and stem rot	*Sclerotinia sclerotiorum*	[133]
*Streptomyces* sp.	Sweet pea	Powdery mildew	*Oidium* sp.	[134]
*S. vinaceusdrappus*	Rice	Blast	*Curvularia oryzae*, *Pyricularia oryzae*, *Bipolaris oryzae*, *Fusarium oxysporum*	[135]
*Streptomyces* sp. RO3	Lemon fruit	Green mold and sour rot	*Penicillium digitatum*, *Geotrichum candidum*	[136]
*S. spororaveus* RDS28	Many	Collar or root rot, stalk rot, leaf spots, and gray mold rot or botrytis blight	*Rhizoctonia solani*, *Fusarium solani*, *Fusarium verticillioides*, *Alternaria alternata*, *Botrytis cinerea*	[137]
*S. toxytricini* vh6	Tomato	Root rot	*Rhizoctonia solani*	[138]
*Streptomyces* spp.	Sugar beet	Root rot	*Rhizoctonia solani*, *Phytophthora drechsleri*	[139]
*Streptomyces* sp.	Chili	Root rot, blight, and fruit rot	*Alternaria brassicae*, *Colletotrichum gloeosporioides*, *Rhizoctonia solani*, *Phytophthora capsica*	[140]
*Streptomyces* sp.	Chili	Wilt	*Fusarium oxysporum* f. sp. *capsici*	[141]
*Streptomyces* sp.	Ginger	Rhizome rot	*Fusarium oxysporum* f. sp. *zingiberi*	[142]
*Streptomyces* sp. CBE	Groundnut	Stem rot	*Sclerotium rolfsii*	[143]
*Streptomyces* sp.	Tomato	Damping off	*Rhizoctonia solani*	[144]
*Streptomyces* sp.	Tobacco	Brown spot	*Alternaria* spp.	[145]
*S. aurantiogriseus* VSMGT1014	Rice	Sheath blight	*Rhizoctonia solani*	[87]
*S. felleus* YJ1	Oilseed rape	Stem rot	*Scleotinia sclerotiorum*	[146]
*S. vinaceusdrappus* S5MW2	Tomato	Root rot	*Rhizoctonia solani*	[147]
*Streptomyces* sp. CACIS-1.16CA	Many	-	*Curvularia* sp., *Aspergillus niger*, *Helminthosporium* sp., *Fusarium* sp. *Alternaria* sp., *Phytophthora capsici*, *Colletotrichum* sp., and *Rhizoctonia* sp.	[148]
*S. griseus*	Tomato	Wilt	*Fusarium* sp.	[149]
*Streptomyces* sp.	Potato	Silver scurf	*Helminthosporium solani*	[150]
*S. rochei*	Pepper	Root rot	*Phytophthora capsica*	[151]
*Streptomyces* sp.	Maize	Seed fungi	*Aspergillus* sp.	[152]
*S. lydicus* WYEC108	Many	Foliar and root fungal diseases	-	[153,154]
*S. griseoviridis* K61	Many	Root rot and wilt pathogenic fungi	-	
*Streptomyces* sp. YCED9 and WYEC108	Lettuce	Damping off	*Pythium ultimum*, *Sclerotinia homeocarpa*, *Rhizoctonia solani*	[153,155]
*Streptomyces* G10	Banana	Wilt	*Fusarium oxysporum* f. sp. *cubense*	[156]
*S. violaceusniger* YCED9	Turfgrass	Crown/foliar disease	*Rhizoctonia solani*	[157]
*Streptomyces* sp.	Cucurbit	Anthracnose	*Colletotrichum orbiculare*	[19]
*Streptomyces* sp. A1022	Pepper and cherry tomato	Anthracnose	*Colletotrichum gloeosporioides*	[158]
*S. halstedii* K122	Many	-	*Aspergillus fumigatus*, *Mucor hiemalis*, *Penicillium roqueforti*, *Paecilomyces variotii*	[159]
*Streptomyces* sp. MT17		Wood rotting	Different fungi	[160]
*S. lavendulae* HHFA1	Onion	Bacterial rot	*Erwinia* *carotovora* subsp. *carotovora, Burkholderia cepacia*	[161]
*S. coelicolor* HHFA2				
*Streptomyces* sp. 5406	Cotton	Soilborne diseases	Soilborne plant pathogens	[162]
*Streptomyces* sp.	Raspberry	Root rot	*Phytophthora fragariae* var. *rubi*	[163]
*S. albidoflavus*	Tomato	Many	*Alternaria solani*, *A. alternata*, *Colletotrichum gloeosporioides*, *Fusarium oxysporum*, *Fusarium solani*, *Rhizoctonia solani*, *Botrytis cinerea*	[164]
*Streptomyces* sp.	Soybean	Bacterial blight	*Xanthomonas campestris* pv. *glycines*	[165]
*Streptomyces* sp. BSA25 and WRAI	Chickpea	-	*Phytophthora medicaginis*	[166]
*Streptomyces* sp.	Chickpea	Fusarium wilt	*Fusarium oxysporum* f. sp. *ciceri*	[167]
*Streptomyces* sp.	Chickpea	Basal rot	*Macrophomina phaseolina*	[73]
*Streptomyces* sp.	Cucumber	Fusarium wilt	*Fusarium oxysporum*	[168]

**Table 3:** List of *Streptomyces* spp.-based products available in the market worldwide (data collected and modified into a table from [202]).

Commercial Product Name	Organism as Active Substance	Registered as Microbial Pesticide	Targeted Pest/Pathogen/Disease
Actinovate, Novozymes BioAg Inc., USA	*S. lydicus WYEC 108*	Canada, USA	Soilborne diseases, viz. *Pythium*, *Fusarium*, *Phytophthora*, *Rhizoctonia*, and *Verticillium*; foliar diseases such as powdery and downy mildew, *Botrytis* and *Alternaria*, *Postia*, *Geotrichum*, and *Sclerotinia*
Mycostop, Verdera Oy, Finland	*Streptomyces K61*	EU, Canada, USA	Damping off caused by *Alternaria* and *R. solani* and *Fusarium*, *Phytophthora*, and *Pythium* wilt and root diseases
Mykocide KIBC Co. Ltd. South Korea	*S. colombiensis*	South Korea	Powdery mildews, grey mold, brown patch
Safegrow KIBC Co. Ltd. South Korea	*S. kasugaensis*	South Korea	Sheath blight, large patch
Actofit, Astur	*S. avermitilis*	Ukraine	Colorado potato beetle, web mites, other phytophags
Bactophil	*Streptomyces albus*	Ukraine	Seed germination diseases
Bialaphos, Toku-E, USA	*S. hygroscopicus*, *S. viridochromogenes*	USA	Herbicide
Incide SP, Sri Biotech Laboratories India Ltd., India	*S. atrovirens*	India	Insecticide
Actin, Sri Biotech Laboratories India Ltd., India	*S. atrovirens*	India	Fungicide

**Table 4:** List of active substances derived from *Streptomyces* spp. (in bold) and registered as commercial products in different geographical areas (data collected and modified into a table from [193,203,204,205]).

Biocontrol Metabolite (Bold) and Commercial Names	Organism	Country	Targeted Pathogen/Disease
**Blasticidin-S** BLA-S	*S. griseochromogenes*	USA	Rice blast (*Pyricularia oryzae*)
**Kasugamycin** Kasumin, Kasurab	*S. kasugaensis*	Ukraine	Leaf spot in sugar beet and celery (*Cercospora* spp.) and scab in pears and apples (*Venturia* spp.), soybean root rot (*Phytophthora sojae*)
**Streptomycin** Agrimycin, Paushak, Cuprimicin 17, AAstrepto 17, AS-50, Dustret, Cuprimic 100 and 500	*S. griseus*	India, USA, New Zealand, China, Ukraine, Canada	Bacterial rots, canker, and other bacterial diseases, *Xanthomonas oryzae*, *Xanthomonas citri*, and *Pseudomonas tabaci* of pome fruit, stone fruit, citrus, olives, vegetables, potatoes, tobacco, cotton, and ornamentals
**Phytomycin** Mycoshield, Cuprimic 100 and 500, Mycoject	*S. rimosus*	-	Fire blight (*Erwinia amylovora*) and diseases caused by *Pseudomonas* and *Xanthomonas* sp. and mycoplasma-like organisms
**Validamycin** Validacin, Valimun, Dantotsupadanvalida, Mycin Hustler, Valida, Sheathmar	*S. hygroscopicus*	-	*Rhizoctonia solani* and other *Rhizoctonia* in rice, potatoes, vegetables, strawberries, tobacco, ginger, cotton, rice, sugar beet, etc.
**Polyoxorim** Endorse, PolyoxinZ, Stopit, Polyoxin AL and Z, Polybelin	*S. cacaoi* var. *asoensis*	-	Plant pathogenic fungi, *Sphaerotheca* spp. and other powdery mildews, *Botrytis cinerea*, *Sclerotinia sclerotiorum*, *Corynespora melonis*, *Cochliobolus miyabeanus*, *Alternaria alternate* and other species in vines, apples, pears, vegetables, and ornamentals. Rice sheath blight (*R. solani*), apple, pear canker, and *Helminthosporium* in rice; also inhibits cell wall biosynthesis and causes abnormal germ tube swelling of spores and hyphal tips, rendering fungus nonpathogenic
**Natamycin** Delvolan	*S. natalensis* and *S. chattanoogensis*	-	Basal rots on daffodils and ornamentals caused by *Fusarium oxysporum*
**Abamectin (Avermectins)** Agri-Meck Avid, Clinch, Dynamec, Vertimec, Abacide, Abamex, Vapcomic, Vibamec, Agromec, Belpromec, Vamectin 1.8 EC, Vivid and many others	*S. avermitilis*	European Union, Worldwide	Mites, leaf miners, suckers, beetles, fire ants, and other insects in ornamentals, cotton, citrus, pome and nut fruit, vegetables
**Polynactin** Mitecidin	*S. aureus*	Japan	Spider mites (*Tetranychus cinnabarinus*), two-spotted mite (*Tetranychus urticae*), European red mite (*Panonychus ulmi*) in orchard fruit trees
**Milbemycine** Milbeknock, Koromite, Mesa, Ultiflora and Matsuguard	*S. hygroscopicus* subsp. *aureolacrimosus*	-	Citrus red mites, Kanzawa spider mites, and leaf miners in citrus, tea, eggplant

**Table 5:** Technical formulations of a set of *Streptomyces*-based products available on international markets, their indications of use, and their inoculation methods. The table has been prepared according to the information given on the labels of the respective products.

Technical Formulation	Commercial Example	Microbial Biocontrol Agent(s)	Indications	Inoculation Method
Granules (G)	Micosat F UNO, CCS Aosta Srl	*Streptomyces* sp. strain SB14	Transplants mortality, plant growth promoter	Soil application as dry granules
Wettable Granules (WG)	Micosat F MO, CCS Aosta Srl	*Streptomyces* spp. strains SA51; SB14 and SL81	Soil bioremediation in viticulture and vegetable production	Soil application as microbial suspension in water
Wettable Powder (WP)	Mykostop, Verdera Oy	*Streptomyces griseoviridis* strain K61	Damping-off fungi, *Phytophthora* spp.	Drip irrigation, cutting/bulb soaking.
Soluble Powder (SP)	Actinovate, Novozymes BioAg Inc.	*Streptomyces lydicus* WYEC 108	Soilborne fungi, powdery mildews, gray moulds	Soil drench, transplants root dipping, foliar sprays, bulb soaking.
Slurry (SE)	-	*Streptomyces* spp. consortium	Bioremediation of organic and inorganic soil pollutants	Soil application as diluted slurry

## References

[1] Smith S., Read D. (1997). Mycorrhizal Symbiosis.

[2] Smith S.E., Smith A.F., Jakobsen I. (2003). Mycorrhizal fungi can dominate phosphate supply to plants irrespective of growth responses. Plant Physiol..

[3] Brader G., Compant S., Mitter B., Trognitz F., Sessitsch A. (2014). Metabolic potential of endophytic bacteria. Curr. Opin. Biotechnol..

[4] Okon Y., Labandera-González C. (1994). Agronomic applications of *Azospirillum*: An evaluation of 20 years of worldwide field inoculation. Soil Biol. Biochem..

[5] Glick B.R., Patten C.L., Holguin G., Penrose D.M. (1999). Biochemical and Genetic Mechanisms Used by Plant Growth Promoting Bacteria.

[6] Dias M.P., Bastos M.S., Xavier V.B., Cassel E., Astarita L.V., Santarém E.R. (2017). Plant growth and resistance promoted by *Streptomyces* spp. in tomato. Plant Physiol. Biochem..

[7] Viaene T., Langendries S., Beirinckx S., Maes M., Goormachtig S. (2016). *Streptomyces* as a plant’s best friend?. FEMS Microbiol. Ecol..

[8] Sturz A.V., Nowak J. (2000). Endophytic communities of rhizobacteria and the strategies required to create yield enhancing associations with crops. Appl. Soil Ecol..

[9] Smith S.E., Read D. (2008). Mycorrhizal Symbiosis.

[10] Bertram R., Schlicht M., Mahr K., Nothaft H., Saier M.H., Titgemeyer F. (2004). In silico and transcriptional analysis of carbohydrate uptake systems of *Streptomyces coelicolor* A3(2). J. Bacteriol..

[11] Chater K.F., Biró S., Lee K.J., Palmer T., Schrempf H. (2010). The complex extracellular biology of *Streptomyces*. FEMS Microbiol. Rev..

[12] Thompson B.J., Widdick D.A., Hicks M.G., Chandra G., Sutcliffe I.C., Palmer T., Hutchings M.I. (2010). Investigating lipoprotein biogenesis and function in the model Gram-positive bacterium *Streptomyces coelicolor*. Mol. Microbiol..

[13] Seipke R.F., Kaltenpoth M., Hutchings M.I. (2012). *Streptomyces* as symbionts: An emerging and widespread theme?. FEMS Microbiol. Rev..

[14] Pangesti N., Pineda A., Pieterse C., Dicke M., Van Loon J.J.A. (2013). Two-way plant mediated interactions between root-associated microbes and insects: From ecology to mechanisms. Front. Plant Sci..

[15] Kupferschmied P., Maurhofer M., Keel C. (2013). Promise for plant pest control: Root-associated pseudomonads with insecticidal activities. Front. Plant Sci..

[16] Baker R. (1991). Diversity in biological control. Crop Prot..

[17] Benhamou N., Kloepper J.W., Quadt-Hallman A., Tuzun S. (1996). Induction of defense-related ultrastructural modifications in pea root tissues inoculated with endophytic bacteria. Plant Physiol..

[18] Varma A., Verma S., Sahay N., Bütehorn B., Franken P. (1999). *Piriformospora indica*, a cultivable plant-growth-promoting root endophyte. Appl. Environ. Microbiol..

[19] Shimizu M., Yazawa S., Ushijima Y.A. (2009). Promising strain of endophytic *Streptomyces* sp. for biological control of cucumber anthracnose. J. Gen. Plant. Pathol..

[20] Sardi P., Saracchi M., Quaroni S., Petrolini B., Borgonovi G.E., Merli S. (1992). Isolation of endophytic *Streptomyces* strains from surface-sterilized roots. Appl. Environ. Microbiol..

[21] Shimizu M., Nakagawa Y., Sato Y., Furumai T., Igarashi Y., Onaka H., Yoshida R., Kunoh H. (2000). Studies on endophytic actinomycetes (I) *Streptomyces* sp. isolated from rhododendron and its antifungal activity. J. Gen. Plant Pathol..

[22] Nishimura T., Meguro A., Hasegawa S., Nakagawa Y., Shimizu M., Kunoh H. (2002). An endophytic actinomycetes, *Streptomyces* sp. AOK-30, isolated from mountain laurel and its antifungal activity. J. Gen. Plant Pathol..

[23] Castillo U., Harper J.K., Strobel G.A., Sears J., Alesi K., Ford E., Lin J., Hunter M., Maranta M., Ge H. (2003). Kakadumycins, novel antibiotics from *Streptomyces* sp. NRRL 30566, an endophyte of *Grevillea pteridifolia*. FEMS Microbiol. Lett..

[24] Coombs J.T., Franco C.M.M. (2003). Isolation and identification of actinobacteria from surface-sterilized wheat roots. Appl. Environ. Microbiol..

[25] Tian X.L., Cao L.X., Tan H.M., Zeng Q.G., Jia Y.Y., Han W.Q., Zhou S.N. (2004). Study on the communities of endophytic fungi and endophytic actinomycetes from rice and their antipathogenic activities in vitro. World J. Microbiol. Biotechnol..

[26] Kunoh H. (2002). Endophytic actinomycetes: Attractive biocontrol agents. J. Gen. Plant Pathol..

[27] Coombs J.T., Michelsen P.P., Franco C.M.M. (2004). Evaluation of endophytic actinobacteria as antagonists of *Gaeumannomyces graminis* var. *tritici* in wheat. Biol. Control..

[28] Meguro A., Ohmura Y., Hasegawa S., Shimizu M., Nishimura T., Kunoh H. (2006). An endophytic actinomycete, *Streptomyces* sp. MBR-52, that accelerates emergence and elongation of plant adventitious roots. Actinomycetologica.

[29] Rothrock C.S., Gottlieb D. (1984). Role of antibiosis in antagonism of *Streptomyces hygroscopicus* var. *geldanus* to *Rhizoctonia solani* in soil. Can. J. Microbiol..

[30] Xiao K., Kinkel L.L., Samac D.A. (2002). Biological control of *Phytophthora* root rots on *alfalfa* and soybean with *Streptomyces*. Biol. Control..

[31] El-Tarabily K.A., Sivasithamparam K. (2006). Non-streptomycete actinomycetes as biocontrol agents of soil-borne fungal plant pathogens and as plant growth promoters. Soil Biol. Biochem..

[32] Kämpfer P., Dworkin M., Falkow S., Rosenberg E., Schleifer K.H., Stackebrandt E. (2006). The family Streptomycetaceae, part I: Taxonomy. The Prokaryotes: A Handbook on the Biology of Bacteria.

[33] Chater K.F., Losick R., Shapiro L. (1984). Morphological physiological differentiation in *Streptomyces*. Microbial Development.

[34] Labeda D.P. (2011). Multilocus sequence analysis of phytopathogenic species of the genus *Streptomyces*. Int. J. Syst. Evol. Microbiol..

[35] Kumar V., Kumar A., Pandey K.D., Roy B.K. (2014). Isolation and characterization of bacterial endophytes from the roots of *Cassia tora* L.. Ann. Microbiol..

[36] Marella S. (2014). Bacterial endophytes in sustainable crop production: Applications, recent developments and challenges ahead. Int. J. Life Sci. Res..

[37] Strobel G.A., Hess W.M., Ford E., Sidhu R.S.W., Yang X. (1996). Taxol from fungal endophytes and the issue of biodiversity. J. Ind. Microbiol..

[38] Strobel G.A., Long D.M. (1998). Endophytic microbes embody pharmaceutical potential. Am. Soc. Microbiol. News.

[39] Watve M., Tickoo R., Jog M., Bhole B. (2001). How many antibiotics are produced by the genus *Streptomyces*?. Arch. Microbiol..

[40] Schrey S.D., Tarkka M.T. (2008). Friends and foes: Streptomycetes as modulators of plant disease and symbiosis. Antonie Leeuwenhoek.

[41] Hibbing M., Fuqua C., Parsek M.R., Peterson S.B. (2010). Bacterial competition: Surviving and thriving in the microbial jungle. Nat. Rev. Microbiol..

[42] Cornforth D.M., Foster K.R. (2013). Competition sensing: The social side of bacterial stress responses. Nat. Rev. Microbiol..

[43] Citron C.A., Barra L., Wink J., Dickschat J.S. (2015). Volatiles from nineteen recently genome sequenced actinomycetes. Org. Biomol. Chem..

[44] Gerber N.N., Lechevalier H.A. (1965). Geosmin, an earthly-smelling substance isolated from actinomycetes. Appl. Microbiol..

[45] Polak E.H., Provasi J. (1992). Odor sensitivity to geosmin enantiomers. Chem. Sens..

[46] Rosenzweig N., Maheshwari D.K. (2014). The importance and application of bacterial diversity in sustainable agricultural crop production systems. Bacterial Diversity in Sustainable Agriculture.

[47] Hopwood D. (2007). Streptomyces in Nature and Medicine: The Antibiotic Makers.

[48] Audrain B., Farag M.A., Choong-Min R., Jean-Marc G. (2015). Role of bacterial volatile compounds in bacterial biology. FEMS Microbiol. Rev..

[49] Ngoc D.P., Eun-Hee L., Seon-Ha C., Yongdeok C., Hyejin S., Ahjeong S. (2016). Bacterial Community Structure Shifted by Geosmin in Granular Activated Carbon System of Water Treatment Plants. J. Microbiol. Biotechnol..

[50] Isaac S. (1992). Fungal-Plant Interactions.

[51] Redlin S.C., Carris L.M., Bills G.F. (1996). Endophytic Fungi in Grasses and Woody Plants: Systemics, ecology and evolution. Isolation and Analysis of Endophytic Fungal Communities from Woody Plants.

[52] Baldani J.I., Olivares F.L., Hemerly A.S., Reis F.B., Oliveira A.L.M., Baldani V.D.L., Goi S.R., Reis V.M., Dobereiner J., Elmerich C., Konderosi A., Newton W.E. (1997). Nitrogen-fixing endophytes: Recent advances in the association with graminaceous plants grown in the tropics. Biological Nitrogen Fixation for the 21st Century.

[53] Azevedo J.L., Meloj I.S., Azevedo L. (1998). Microrganismos endofíticos. Ecologia Microbiana.

[54] Saleem M., Law A.D., Moe L.A. (2016). Nicotiana roots recruit rare rhizosphere taxa as major root-inhabiting microbes. Microb. Ecol..

[55] Figueiredo M.V.B., Seldin L., de Araujo F.F., Mariano R.L.R., Maheshwari D. (2010). Plant Growth Promoting Rhizobacteria: Fundamentals and Applications. Plant Growth and Health Promoting Bacteria.

[56] Sousa J.A.A., Olivares F.L. (2016). Plant growth promotion by streptomycetes: Ecophysiology, mechanisms and applications. Chem. Biol. Technol. Agric..

[57] Vardharajula S., Shaik Zulfikar A., Vurukonda S.S.K.P., Shrivastava M. (2017). Plant growth promoting endophytes and their interaction with plants to alleviate abiotic stress. Curr. Biotechnol..

[58] Benson D.R., Silvester W.B. (1993). Biology of *Frankia* strains, actinomycete symbionts of actinorhizal plants. Microbiol. Rev..

[59] Bradbury J.F. (1986). Guide to Plant Pathogenic Bacteria.

[60] Mundt J.O., Hinckle N.F. (1976). Bacteria within ovules and seeds. Appl. Environ. Microbiol..

[61] Sathya A., Vijayabharathi R., Gopalakrishnan S. (2017). Plant growth-promoting actinobacteria: A new strategy for enhancing sustainable production and protection of grain legumes. 3 Biotech.

[62] Bulgarelli D., Schlaeppi K., Spaepen S., Ver E., Van Themaat L., Schulze-Lefert P. (2013). Structure and functions of the bacterial microbiota of plants. Annu. Rev. Plant Biol..

[63] Massalha H., Korenblum E., Tholl D., Aharoni A. (2017). Small molecules below-ground: The role of specialized metabolites in the rhizosphere. Plant J..

[64] Bais H.P., Weir T.L., Perry L.G., Gilroy S., Vivanco J.M. (2006). The role of root exudates in rhizosphere interactions with plants and other organisms. Ann. Rev. Plant Biol..

[65] Jog R., Nareshkumar G., Rajkumar S., Gopalakrishnan S., Sathya A., Vijayabharathi R. (2016). Enhancing soil health and plant growth promotion by actinomycetes. Plant Growth Promoting Actinobacteria.

[66] Meschke H., Schrempf H. (2010). *Streptomyces lividans* inhibits the proliferation of the fungus *Verticillium dahliae* on seeds and roots of *Arabidopsis thaliana*. Microb. Biotechnol..

[67] Palaniyandi S.A., Yang S.H., Damodharan K., Suh J.W. (2013). Genetic and functional characterization of culturable plant-beneficial actinobacteria associated with yam rhizosphere. J. Basic Microbiol..

[68] Rungin S., Indananda C., Suttiviriya P., Kruasuwan W., Jaemsaeng R., Thamchaipenet A. (2012). Plant growth enhancing effects by a siderophore producing endophytic streptomycete isolated from a Thai jasmine rice plant (*Oryza sativa* L. cv. KDML105). Antonie Leeuwenhoek.

[69] Qin S., Xing K., Jiang J.H., Xu L.H., Li W.J. (2011). Biodiversity, bioactive natural products and biotechnological potential of plant-associated endophytic actinobacteria. Appl. Microbiol. Biotechnol..

[70] Coombs J.T., Franco C.M. (2003). Visualization of an endophytic *Streptomyces* species in wheat seed. Appl. Environ. Microbiol..

[71] Shimizu M., Maheshwari D.K. (2011). Endophytic actinomycetes: Biocontrol agents and growth promoters. Bacteria in Agrobiology: Plant Growth Responses.

[72] Franco-Correa M., Quintana A., Duque C., Suarez C., Rodriguez M.X., Barea J.M. (2010). Evaluation of actinomycete strains for key traits related with plant growth-promotion and mycorrhiza helping activities. Appl. Soil Ecol..

[73] Gopalakrishnan S., Vadlamudi S., Bandikinda P., Sathya A., Vijayabharathi R., Rupela O., Kudapa H., Katta K., Varshney R.K. (2014). Evaluation of *Streptomyces* strains isolated from herbal vermicompost for their plant growth-promotion traits in rice. Microbiol. Res..

[74] Gopalakrishnan S., Srinivas V., Alekhya G., Prakash B., Kudapa H., Varshney R.K. (2015). Evaluation of *Streptomyces* sp. obtained from herbal vermicompost for broad spectrum of plant growth-promoting activities in chickpea. Org. Agric..

[75] Tokala R., Strap J., Jung C.M., Crawford D.L., Salove M.H., Deobald L.A., Bailey J.F., Morra M.J. (2002). Novel plant microbe rhizosphere interaction involving *Streptomyces lydicus* WYEC 108 and the pea plant (*Pisum sativum*). Appl. Environ. Microbiol..

[76] Nimnoi P., Pongsilp N., Lumyong S. (2014). Co-inoculation of soybean (*Glycine max*) with actinomycetes and *Bradyrhizobium japonicum* enhances plant growth, nitrogenase activity and plant nutrition. J. Plant Nutr..

[77] Abd-Alla M.H., El-Sayed E.A., Rasmey A.M. (2013). Indole-3-acetic acid (IAA) production by *Streptomyces atrovirens* isolated from rhizospheric soil in Egypt. J. Biol. Earth Sci..

[78] El-Tarabily K.A. (2008). Promotion of tomato (*Lycopersicon esculentum* Mill.) plant growth by rhizosphere competent 1-aminocyclopropane-1-carboxylic acid deaminase-producing streptomycete actinomycetes. Plant Soil.

[79] Khamna S., Yokota A., Peberdy J.F., Lumyong S. (2010). Indole-3-acetic acid production by *Streptomyces* sp. isolated from some Thai medicinal plant rhizosphere soils. Eur. Asia J. Biosci..

[80] Verma V.C., Singh S.K., Prakash S. (2011). Bio-control and plant growth-promotion potential of siderophore producing endophytic *Streptomyces* from *Azadirachta indica* A. Juss. J. Basic Microbiol..

[81] Lin L., Xu X. (2013). Indole-3-acetic acid production by endophytic *Streptomyces* sp. En-1 isolated from medicinal plants. Curr. Microbiol..

[82] Tsavkelova E.A., Klimova S.Y., Cherdyntseva T.A., Netrusov A.I. (2006). Microbial producers of plant growth-stimulators and their practical use: A review. Appl. Biochem. Microbiol..

[83] Nascimento F.X., Rossi M.J., Soares C.R., McConkey B.J., Glick B.R. (2014). New insights into 1-aminocyclopropane-1-carboxylate (ACC) deaminase phylogeny, evolution and ecological significance. PLoS ONE.

[84] Goudjal Y., Zamoum M., Meklat A., Sabaou N., Mathieu F., Zitouni A. (2015). Plant growth-promoting potential of endosymbiotic actinobacteria isolated from sand truffles (*Terfezia leonis* Tul.) of the Algerian Sahara. Ann. Microbiol..

[85] Rashad F.M., Fathya H.M., El-Zayata A.S., Elghonaimy A.M. (2015). Isolation and characterization of multifunctional *Streptomyces* species with antimicrobial, nematicidal and phytohormone activities from marine environments in Egypt. Microbiol. Res..

[86] Harikrishnan H., Shanmugaiah V., Balasubramanian N. (2014). Optimization for production of indole acetic acid (IAA) by plant growth-promoting *Streptomyces* sp. VSMGT1014 isolated from rice rhizosphere. Int. J. Curr. Microbiol. Appl. Sci..

[87] Harikrishnan H., Shanmugaiah V., Balasubramanian N., Sharma M.P., Kotchoni S.O. (2014). Antagonistic potential of native strain *Streptomyces aurantiogriseus* VSMGT1014 against Sheath Blight of rice disease. World J. Microbiol. Biotechnol..

[88] Rafik E., Rahal E., Ahmed L. (2014). Isolation and screening of actinomycetes strains producing substances plant growth-promoting. Indo-Am. J. Agric. Vet. Sci..

[89] Gopalakrishnan S., Vadlamudi S., Apparla S., Bandikinda P., Vijayabharathi R., Bhimineni R.K., Rupela O. (2013). Evaluation of *Streptomyces* spp. for their plant growth-promotion traits in rice. Can. J. Microbiol..

[90] Lee J., Postmaster A., Soon H.P., Keast D., Carson K.C. (2012). Siderophore production by Actinomycetes isolates from two soil sites in Western Australia. Biometals.

[91] Jog R., Nareshkumar G., Rajkumar S. (2012). Plant growth promoting potential and soil enzyme production of the most abundant *Streptomyces* spp. from wheat rhizosphere. J. Appl. Microbiol..

[92] Ghodhbane-Gtari F., Essoussi I., Chattaoui M., Chouaia B., Jaouani A., Daffonchio D., Boudabous A., Gtari M. (2010). Isolation and characterization of non-*Frankia* actinobacteria from root nodules of *Alnus glutinosa*, *Casuarina glauca* and *Elaeagnus angustifolia*. Symbiosis.

[93] Aldesuquy H.S., Mansour F.A., Abo-Hamed S.A. (1998). Effect of the culture filtrates of *Streptomyces* on growth and productivity of wheat plants. Folia Microbiol..

[94] Igarashi Y., Iida T., Yoshida R., Furumai T. (2002). Pteridic acids A and B, novel plant growth-promoters with auxin-like activity from *Streptomyces hygroscopicus* TP-A0451. J. Antibiot..

[95] El-Tarabily K.A., Nassar A.H., Hardy G.E.S.J., Sivasithamparam K. (2009). Plant growth-promotion and biological control of *Pythium aphanidermatum*, a pathogen of cucumber, by endophytic actinomycetes. J. Appl. Microbiol..

[96] Gopalakrishnan S., Srinivas V., Vidya M.S., Rathore A. (2013). Plant growth-promoting activities of *Streptomyces* spp. in sorghum and rice. SpringerPlus.

[97] Dochhil H., Dkhar M.S., Barman D. (2013). Seed germination enhancing activity of endophytic Streptomyces isolated from indigenous ethno-medicinal plant Centella asiatica. Int. J. Pharm. Biol. Sci..

[98] El-Tarabily K.A., Hardy G.E.S.J., Sivasithamparam K. (2010). Performance of three endophytic actinomycetes in relation to plant growth promotion and biological control of *Pythium aphanidermatum*, a pathogen of cucumber under commercial field production conditions in the United Arab Emirates. Eur. J. Plant Pathol..

[99] Golinska P., Magdalena W., Gauravi A., Dnyaneshwar R., Hanna D., Mahendra R. (2015). Endophytic actinobacteria of medicinal plants: Diversity and bioactivity. Antonie Leeuwenhoek.

[100] Goodfellow M., Simpson K.E. (1987). Ecology of streptomycetes. Front. Appl. Microbiol..

[101] Suzuki S., Yamamoto K., Okuda T., Nishio M., Nakanishi N., Komatsubara S. (2000). Selective isolation and distribution of *Actinomadura rugatobispora* strains in soil. Actinomycetologica.

[102] Alam M., Dahrni S., Khaliq A., Srivastava S.K., Samad A., Gupta M.K. (2012). A promising strain of *Streptomyces* sp. with agricultural traits for growth-promotion and disease management. Indian J. Exp. Microbiol..

[103] Inbar E., Green S.J., Hadar Y., Minz D. (2005). Competing factors of compost concentration and proximity to root affect the distribution of Streptomycetes. Microb. Ecol..

[104] Manulis S., Epstein E., Shafrir H., Lichter A., Barash I. (1994). Biosynthesis of indole-3-acetic acid via the indole-3-acetamide pathway in *Streptomyces* spp.. Microbiology.

[105] Reddy K.R.K., Jyothi G., Sowjanya Ch., Kusumanjali K., Malathi N., Reddy K.R.N., Subramaniam G. (2016). Plant Growth-Promoting Actinomycetes: Mass Production, Delivery systems, and commercialization. Plant Growth Promoting Actinobacteria.

[106] El-Sayed M.A., Valadon L.R.G., El-Shanshoury A. (1987). Biosynthesis and metabolism of indole-3-acetic acid in Streptomyces mutabilis and Streptomyces atroolivaceus. Microbiol. Lett..

[107] El-Shanshoury A.R. (1991). Biosynthesis of indole-3-acetic acid in Streptomyces atroolivaceus and its changes during spore germination and mycelial growth. Microbiol. Lett..

[108] Sellstedt A., Richau K.H. (2013). Aspects of nitrogen-fixing Actinobacteria, in particular free-living and symbiotic *Frankia*. FEMS Microbiol. Lett..

[109] Fiedler H., Krastel P., Müller J., Gebhardt K., Zeeck A. (2001). Enterobactin: The characteristic catecholate siderophore of Enterobacteriaceae is produced by *Streptomyces* species. FEMS Microbiol. Lett..

[110] Van Driesche R.G., Bellows T.S. (1996). Biological Control.

[111] Procópio R.E., Silva I.R., Martins M.K., Azevedo J.L., Araújo J.M. (2012). Antibiotics produced by *Streptomyces*. Braz. J. Infect. Dis..

[112] Whipps J.M. (2001). Microbial interactions and biocontrol in the rhizosphere. J. Exp. Bot..

[113] Défago G. (1993). 2,4-Diacetylphloroglucinol, a promising compound in biocontrol. Plant Pathol..

[114] De Souza J.T., de Boer M., de Waard P., van Beek T.A., Raaijmakers J.M. (2003). Biochemical, genetic, and zoosporicidal properties of cyclic lipopeptide surfactants produced by *Pseudomonas fluorescens*. Appl. Environ. Microbiol..

[115] Kim B.S., Moon S.S., Hwang B.K. (1999). Isolation, identification and antifungal activity of a macrolide antibiotic, oligomycin A, produced by *Streptomyces libani*. Can. J. Bot..

[116] Compant S., Duffy B., Nowak J., Clément C., Barka E.A. (2005). Use of plant growth-promoting bacteria for biocontrol of plant diseases: Principles, mechanisms of action and future prospects. Appl. Environ. Microbiol..

[117] Pridham T.G., Lindenfesler L.A., Shotwell O.L., Stodola F.H., Bendict R.G., Foley C., Jackson R.W., Zaumeyr J.W., Preston W.H., Mitchell J.W. (1956). Antibiotics against plant disease, I. Laboratory and green house survey. Phytopathology.

[118] Pridham T.G., Shotwell O.L., Stodola F.H., Lindenfesler L.S., Bendict R.G., Jackson R.W. (1956). Antibiotics against plant disease. II. Effective agents produced by *Streptomyces cinnamomeous forma azacoluta* F. Nov. Phytopathol..

[119] Reddy G.S., Rao A.S. (1971). Antagonism of soil actinomycetes to some soil borne plant pathogenic fungi. Indian Phytopathol..

[120] Rothrock C.S., Gottlieb D. (1981). Importance of antibiotic production in antagonism of selected *Streptomyces* species to two soil-borne plant pathogens. J. Antibiot..

[121] Papavizas G.C., Sutherland E.D. (1991). Evaluation of oospore hyper-Parasites for the control of *Phytophthora* crown rot of pepper. J. Phytopathol..

[122] El–Tarabily K.A., Soliman M.H., Nassar A.H., Al–Hassani H.A., Sivasithamparam K., McKenna F., Hardy G.E.S.J. (2000). Biocontrol of *Sclerotinia* minor using a chitinolytic bacterium and actinomycetes. Plant Pathol..

[123] Getha K., Vikineswary S. (2002). Antagonistic effects of *Streptomyces violaceusniger* strain G10 on *Fusarium oxysporum* f. sp. *cubense* race 4: Indirect evidence for the role of antibiosis in the antagonistic process. J. Ind. Microbiol. Biotechnol..

[124] Rhee K.H. (2003). Purification and identification of an antifungal agent from *Streptomyces* sp. KH-614 antagonistic to rice blast fungus, *Pyricularia oryzae*. J. Microbiol. Biotechnol..

[125] Woo J.H., Kamei Y. (2003). Antifungal mechanism of an anti–pythium protein (SAP) from the marine bacterium *Streptomyces* sp. strain AP77 is specific for *P. porphyrae*, a causative agent of red rot disease in *Porhyra* spp.. Appl. Microbiol. Biotechnol..

[126] Cao L., Qiu Z., You J., Tan H., Zhou S. (2004). Isolation and characterization of endophytic *Streptomyces* strains from surface–sterilized tomato (*Lycopersicon esculentum*) roots. Lett. Appl. Microbiol..

[127] Joo G.J. (2005). Production of an anti-fungal substance for biological control of *Phytophthora capsici* causing phytophthora blight in red–peppers by *Streptomyces halstedii*. Biotechnol. Lett..

[128] Liang J.F., Xue Q.H., Niu X.L., Li Z.B. (2005). Root colonization and effects of seven strains of actinomycetes on leaf PAL and PPO activities of capsicum. Acta Bot. Boreal-Occident Sin..

[129] Shekhar N., Bhattacharya D., Kumar D., Gupta K.R. (2006). Biocontrol of wood-rotting fungi with *Streptomyces violaceusniger* XL-2. Can. J. Microbiol..

[130] Heng J.L.S., Md Shah U.K., Abdul Rahman N.A., Shaari K., Halizah H. (2006). *Streptomyces ambofaciens* S2—A potential biological control agent for *Colletotrichum gleosporioides* the causal agent for anthracnose in red chilli fruits. J. Plant Pathol. Microbiol..

[131] Errakhi R., Bouteau F., Lebrihi A., Barakate M. (2007). Evidences of biological control capacities of *Streptomyces* spp. against *Sclerotium rolfsii* responsible for damping-off disease in sugar beet (*Beta vulgaris* L.). World J. Microbiol. Biotechnol..

[132] Prapagdee B., Kuekulvong C., Mongkolsuk S. (2008). Antifungal potential of extracellular metabolites produced by *Streptomyces hygroscopicus* against phytopathogenic fungi. Int. J. Biol. Sci..

[133] Baniasadi F., Bonjar G.H.S., Baghizadeh A., Nick A.K., Jorjandi M., Aghighi S., Farokhi P.R. (2009). Biological control of *Sclerotinia sclerotiorum*, causal agent of sunflower head and stem rot disease, by use of soil borne actinomycetes isolates. Am. J. Agric. Biol. Sci..

[134] Sangmanee P., Bhromsiri A., Akarapisan A. (2009). The potential of endophytic actinomycetes, (*Streptomyces* sp.) for the biocontrol of powdery mildew disease in sweet pea (*Pisum sativum*). Asian J. Food Agric. Ind..

[135] Ningthoujam D.S., Sanasam S., Tamreihao K., Nimaichand S. (2009). Antagonistic activities of local actinomycete isolates against rice fungal pathogens. Afr. J. Microbiol. Res..

[136] Maldonado M.C., Orosco C.E., Gordillo M.A., Navarro A.R. (2010). In vivo and in vitro antagonism of *Streptomyces* sp. RO3 against *Penicillium digitatum* and *Geotrichum candidum*. Afr. J. Microbiol. Res..

[137] Al–Askar A.A., Abdul Khair W.M., Rashad Y.M. (2011). In vitro antifungal activity of *Streptomyces spororaveus* RDS28 against some phytopathogenic fungi. Afr. J. Agric. Res..

[138] Patil H.J., Srivastava A.K., Singh D.P., Chaudhari B.L., Arora D.K. (2011). Actinomycetes mediated biochemical responses in tomato (*Solanum lycopersicum*) enhances bioprotection against *Rhizoctonia solani*. Crop Prot..

[139] Karimi E., Sadeghi A., Dehaji P.A., Dalvand Y., Omidvari M., Nezhad M.K. (2012). Biocontrol activity of salt tolerant *Streptomyces* isolates against phytopathogens causing root rot of sugar beet. Biocontrol. Sci. Technol..

[140] Srividya S., Thapa A., Bhat D.V., Golmei K., Nilanjan D. (2012). *Streptomyces* sp. 9p as effective biocontrol against chili soilborne fungal phytopathogens. Eur. J. Exp. Biol..

[141] Saengnak V., Chaisiri C., Nalumpang S. (2013). Antagonistic *Streptomyces* species can protect chili plants against wilt disease caused by *Fusarium*. J. Agric. Technol..

[142] Manasa M., Yashoda K., Pallavi S., Vivek M.N., Onkarappa R., Kekuda T.R.P. (2013). Biocontrol potential of *Streptomyces* species against *Fusarium oxysporum* f. sp. *zingiberi* (causal agent of rhizome rot of ginger). J. Adv. Sci. Res..

[143] Adhilakshmi M., Latha P., Paranidharan V., Balachandar D., Ganesamurthy K., Velazhahan R. (2014). Biological control of stem rot of groundnut (*Arachis hypogaea* L.) caused by *Sclerotium rolfsii* Sacc. with actinomycetes. Arch. Phytopathol. Plant Protect..

[144] Goudjal Y., Toumatiaa O., Yekkour A., Sabaoua N., Mathieuc F., Zitouni A. (2014). Biocontrol of *Rhizoctonia solani* damping–off and promotion of tomato plant growth by endophytic actinomycetes isolated from native plants of Algerian Sahara. Microbiol. Res..

[145] Gao F., Wu U., Wang M. (2014). Identification and antifungal activity of an actinomycete strain against *Alternaria* spp.. Span. J. Agric. Res..

[146] Cheng G., Huang Y., Yang Y., Liu F. (2014). *Streptomyces felleus* YJ1: Potential biocontrol agents against the *sclerotinia* stem rot (*Sclerotinia sclerotiorum*) of oilseed rape. J. Agric. Sci..

[147] Yandigeri M.S., Malviya N., Solanki M.K., Shrivastava P., Sivakumar G. (2015). Chitinolytic *Streptomyces vinaceusdrappus* S5MW2 isolated from Chilika lake, India enhances plant-growth and biocontrol efficacy through chitin supplementation against *Rhizoctonia solani*. World J. Microbiol. Biotechnol..

[148] Zahaed E.M. (2014). Isolation and characterization of soil *Streptomyces* species as potential biological control agents against fungal plant pathogens. World J. Microbiol. Biotechnol..

[149] Anitha A., Rabeeth M. (2009). Control of *Fusarium* wilt of tomato by bioformulation of *Streptomyces griseus* in green house condition. Afr. J. Basic Appl. Sci..

[150] Elson M.K. (1997). Selection of microorganisms for biological control of silver scurf (*Helminthosporium solani*) of potato tubers. Plant Dis..

[151] Ezziyyani M., Requena M.E., Egea-Gilabert C., Candela M.E. (2007). Biological control of *Phytophthora* root rot of pepper using *Trichoderma harzianum* and *Streptomyces rochei* in combination. J. Phytopathol..

[152] Bressan W. (2003). Biological control of maize seed pathogenic fungi by use of actinomycetes. BioControl.

[153] Crawford D.L., Lynch J.M., Whipps J.M., Ousley M.A. (1993). Isolation and characterization of actinomycete antagonists of a fungal root pathogen. Appl. Environ. Microbiol..

[154] Lahdenpera M.L. (1987). The control of *Fusarium* wilt on carnation with a *Streptomyces* preparation. Acta Horticult..

[155] Crawford D.L. (1996). Use of *Streptomyces* Bacteria to Control Plant Pathogens. U.S. Patent.

[156] Getha K., Vikineswary S., Wong W.H., Seki T., Ward A., Goodfellow M. (2005). Evaluation of *Streptomyces* sp. strain g10 for suppression of *Fusarium* wilt and rhizosphere colonization in pot-grown banana plantlets. J. Ind. Microbiol. Biotechnol..

[157] Trejo-Estrada S.R., Sepulveda I.R., Crawford D.L. (1998). In vitro and in vivo antagonism of *Streptomyces violaceusniger* YCED9 against fungal pathogens of turfgrass. World J. Microbiol. Biotechnol..

[158] Kim H.J., Lee E.J., Park S.H., Lee H.S., Chung N. (2014). Biological control of anthracnose (*Colletotrichum gloeosporioides*) in pepper and cherry tomato by *Streptomyces* sp. A1022. J. Agric. Sci..

[159] Frändberg E., Petersson C., Lundgern L.N., Schnürer J. (2000). *Streptomyces halstedii* K122 produces the antifungal compounds bafilomycin B1 and C1. Can. J. Microbiol..

[160] Nagpure A., Choudhary B., Shanti K., Gupta R.K. (2014). Isolation and characterization of chitinolytic *Streptomyces* sp. MT7 and its antagonism towards wood rotting fungi. Ann. Microbiol..

[161] Abdallah M.E., Haroun S.A., Gomah A.A., El-Naggar N.E., Badr H.H. (2013). Application of actinomycetes as biocontrol agents in the management of onion bacterial rot diseases. Arch. Phytopathol. Plant Protect..

[162] Yin S.Y., Chang J.K., Xun P.C. (1965). Studies in the mechanisms of antagonistic fertilizer “5406”. IV. The distribution of the antagonist in soil and its influence on the rhizosphere. Acta Microbiol. Sin..

[163] Valois D., Fayad K., Barasubiye T., Garon M., Dery C., Brzezinski R., Beaulieu C. (1996). Glucanolytic actinomycetes antagonistic to *Phytophthora fragariae* var. *rubi*, the causal agent of raspberry root rot. Appl. Environ. Microbiol..

[164] Haggag W.M., Singer S.M., Mohamed D.E.H.A. (2014). Application of broad-spectrum of marine *Streptomyces albidoflavus* a biofungicide and plant promoting of tomato diseases. Res. J. Pharm. Biol. Chem..

[165] Mingma R., Pathom-aree W., Trakulnaleamsai S., Thamchaipenet A., Duangmal K. (2014). Isolation of rhizospheric and roots endophytic actinomycetes from *Leguminosae* plant and their activities to inhibit soybean pathogen, *Xanthomonas campestris* pv. *glycine*. World J. Microbiol. Biotechnol..

[166] Misk A., Franco C. (2011). Biocontrol of chickpea root rot using endophytic actinobacteria. BioControl.

[167] Gopalakrishnan S., Pandey S., Sharma M., Humayun P., Kiran B.K., Sandeep D., Vidya M.S., Deepthi K., Rupela O. (2011). Evaluation of actinomycete isolates obtained from herbal vermicompost for the biological control of *Fusarium* wilt of chickpea. Crop Prot..

[168] Singh P.P., Shin Y.C., Park C.S., Chung Y.R. (1999). Biological control of *fusarium* wilt of cucumber by chitinolytic bacteria. Phytopathology.

[169] Samac D.A., Willert A.M., McBride M.J., Kinkel L.L. (2003). Effects of antibiotic-producing *Streptomyces* on nodulation and leaf spot in *Alfalfa*. Appl. Soil. Ecol..

[170] Conn V.M., Walker A.R., Franco C.M. (2008). Endophytic actinobacteria induce defense pathways in *Arabidopsis thaliana*. Mol. Plant Microbe Interact..

[171] Bacon C.W., Hinton D.M., Gnanamanickam S.S. (2006). Bacterial endophytes: The endophytic niche, its occupants, and its utility. Plant Associated Bacteria.

[172] Hastuti R.D., Yulin L., Antonius S., Rasti S. (2012). Endophytic *Streptomyces* sp. as Biocontrol Agents of Rice Bacterial Leaf Blight Pathogen (*Xanthomonas oryzae* pv. *oryzae*). HAYATI J. Biosci..

[173] Gupta R., Saxena R.K., Chaturvedi P., Virdi J.S. (1995). Chitinase production by *Streptomyces viridificans*: Its potential in fungal cell wall lysis. J. Appl. Bacteriol..

[174] Suzuki S., Nakanishi E., Ohira T., Kawachi R., Ohnishi Y., Horinouchi S., Nagasawa H., Sakuda S. (2006). Chitinase inhibitor allosamidin is a signal molecule for chitinase production in its producing *Streptomyces*. II. Mechanism for regulation of chitinase production by allosamidin through a two-component regulatory system. J. Antibiot..

[175] Adams D.J. (2004). Fungal cell wall chitinases and glucanases. Microbiology.

[176] Chernin L., Ismailov Z., Haran S., Chet I. (1995). Chitinolytic *Enterobacter agglomerans* antagonistic to fungal plant pathogens. Appl. Environ. Microbiol..

[177] De Boer W., Klein Gunnewiek P.J.A., Kowalchuk G.A., Van Veen J.A. (2001). Growth of Chitinolytic Dune Soil β-Subclass *Proteobacteria* in Response to Invading Fungal Hyphae. Appl. Environ. Microbiol..

[178] Sakuda S., Isogai A., Matsumoto S., Suzuki A. (1987). Search for microbial insect growth regulators II. Allosamidin, a novel insect chitinase inhibitor. J. Antibiot..

[179] Suzuki S., Nakanishi E., Ohira T., Kawachi R., Nagasawa H., Sakuda S. (2006). Chitinase inhibitor allosamidin is a signal molecule for chitinase production in its producing *Streptomyces*. I. Analysis of the chitinase whose production is promoted by allosamidin and growth accelerating activity of allosamidin. J. Antibiot..

[180] Sakuda S., Inoue H., Nagasawa H. (2013). Novel Biological Activities of Allosamidins. Molecules.

[181] Cao L., Qiu Z., You J., Tan H., Zhou S. (2005). Isolation and characterization of endophytic *Streptomyces* antagonists of *Fusarium* wilt pathogen from surface sterilized banana roots. FEMS Microbiol. Lett..

[182] Tan H.M., Cao L.X., He Z.F., Su G.J., Lin B., Zhou S.N. (2006). Isolation of endophytic actinomycetes from different cultivars of tomato and their activities against *Ralstonia solanacearum* in vitro. World J. Microbiol. Biotechnol..

[183] El-Shatoury S., El-Kraly O., El-Kazzaz W., Dewedar A. (2009). Antimicrobial activities of Actinomycetes inhabiting *Achillea fragrantissima* (Family: Compositae). Egypt. J. Nat. Toxins.

[184] Gangwar M., Dogra S., Gupta U.P., Kharwar R.N. (2014). Diversity and biopotential of endophytic actinomycetes from three medicinal plants in India. Afr. J. Microbiol. Res..

[185] El-Tarabily K.A. (2003). An endophytic chitinase-producing isolate of Actinoplanes missouriensis, with potential for biological control of root rot of lupine caused by *Plectosporium tabacinum*. Aust. J. Bot..

[186] Kloepper J.W., Leong J., Teintze M., Schiroth M.N. (1980). Enhanced plant growth by siderophores produced by plant growth promoting rhizobacteria. Nature.

[187] Igarashi Y. (2004). Screening of novel bioactive compounds from plant-associated actinomycetes. Actinomycetolog.

[188] Zhang J., Wang J.D., Liu C.X., Yuan J.H., Wang X.J., Xiang W.S. (2014). A new prenylated indole derivative from endophytic actinobacteria *Streptomyces* sp. neau-D50. Nat. Prod. Res..

[189] Lu C., Shen Y.A. (2003). New macrolide antibiotic with antitumor activity produced by *Streptomyces* sp. CS, a commensal microbe of Maytenus hookeri. J. Antibiot..

[190] Lu C., Shen Y. (2007). A novel ansamycin, naphthomycin k from *Streptomyces* sp.. J. Antibiot..

[191] Igarashi Y., Iida T., Sasaki T., Saito N., Yoshida R., Furumai T. (2002). Isolation of actinomycetes from live plants and evaluation of anti-phytopathogenic activity of their metabolites. Actinomycetolog.

[192] Book A.J., Lewin G.R., McDonald B.R., Takasuka T.E., Doering D.T., Adams A.S., Blodgett J.A.V., Clardy J., Raffa K.F., Fox B.G. (2014). Cellulolytic *Streptomyces* strains associated with herbivorous insects share a phylogenetically linked capacity to degrade lignocelluloses. Appl. Environ. Microbiol..

[193] Copping G.L., Menn J.J. (2000). Biopesticides: A review of their action, applications and efficacy. Pest Manag. Sci..

[194] Craveri R., Giolitti G. (1957). An antibiotic with fungicidal and insecticidal activity produced by *Streptomyces*. Nature.

[195] Kido G.S., Spyhalski E. (1950). Antimycin A, an antibiotic with insecticidal and miticidal properties. Science.

[196] Takahaski N., Suzuki A., Kimura Y., Miyamoto S., Tamura S., Mitsui T., Fukami J. (1968). Isolation, structure and physiological activities of piericidin B, natural insecticide produced by a *Streptomyces*. Agric. Biol. Chem..

[197] Oishi H., Sugawa T., Okutomi T., Suzuki K., Hayashi T., Sawada M., Ando K. (1970). Insecticidal activity of macrotetrolide antibiotics. J. Antibiot..

[198] Box S.J., Cole M., Yeoman G.H. (1973). Prasinons A and B: Potent insecticides from *Streptomyces prasinus*. Appl. Microbiol..

[199] Turner M.J., Schaeffer J.M., Cambell W.C. (1989). Mode of action of ivermectin. Ivermectin and Abamectin.

[200] Bloomquist J.R. (1996). Ion Channels as Targets for Insecticides. Annu. Rev. Entomol..

[201] Sousa S.C., Soares F.A.C., Garrido S.M. (2008). Characterization of Streptomycetes with potential to promote plant growth and biocontrol. Sci. Agric..

[202] Kabaluk J.T., Svircev A.M., Goettel M.S., Woo S.G. (2010). The Use and Regulation of Microbial Pesticides in Representative Jurisdiction Worldwide.

[203] Copping L.G., Duke S.O. (2007). Review-natural products that have been used commercially as crop protection agents. Pest Manag. Sci..

[204] Saxena S. (2014). Microbial metabolites for development of ecofriendly agrochemical. Allelopathy J..

[205] Aggarwal N., Thind S.K., Sharma S., Subramaniam G. (2016). Role of secondary metabolites of Actinomycetes in crop protection. Plant Growth Promoting Actinobacteria.

[206] Tanaka Y., Omura S. (1993). Agro-active compounds of microbial origin. Annu. Rev. Microbiol..

[207] Behal V. (2000). Bioactive products from *Streptomyces*. Adv. Appl. Microbiol..

[208] Alexander M. (1977). Introduction to Soil Microbiology.

[209] Isono K., Nagatsu J., Kobinata K., Sasaki K., Suzuki S. (1965). Studies on polyoxins, antifungal antibiotics. Part I. Isolation and characterization of polyoxins A and B. Agric. Biol. Chem..

[210] Umezawa H., Okami Y., Hashimoto T., Suhara Y., Hamada M., Takeuchi T. (1965). A new antibiotic, kasugamycin. J. Antibiot..

[211] Siddique S., Syed Q., Adnan A., Nadeem M., Irfan M., Qureshi F.A. (2013). Production of avermectin B1b from Streptomyces avermitilis 41445 by batch submerged fermentation. Jundishapur J. Microbiol..

[212] Reddy K.R.K., Jyothi G., Sowjanya Ch., Kusumanjali K., Malathi N., Reddy K.R.N., Subramaniam G. (2016). Plant Growth-Promoting Actinomycetes: Mass Production, Delivery systems, and commercialization. Plant Growth Promoting Actinobacteria.

[213] Subramaniam G., Arumugam S., Rajendran V., Vadlamudi S., Singh D.P., Singh H.B., Prabha R. (2016). Plant Growth Promoting Micorbes for Field Applications. Microbial Inoculants in Sustainable Agriculture Productivity.

[214] Hoddle M., FAO (2005). International Standards for Phytosanitary Measures. Guidelines for the export, shipment, import and release of biological control agents and other beneficial organisms. Second International Symposium on Biological Control of Arthropods.

[215] Rafii F., Crawford D.L., Bleakley B.H., Wang Z. (1988). Assessing the risks of releasing recombinant *Streptomyces* in soil. Microbiol. Sci..

[216] Egan S., Wiener P., Kallifidas D., Wellington E.M. (1998). Transfer of streptomycin biosynthesis gene clusters within streptomycetes isolated from soil. Appl. Environ. Microbiol..

[217] Egan S., Wiener P., Kallifidas D., Wellington E.M. (2001). Phylogeny of *Streptomyces* species and evidence for horizontal transfer of entire and partial antibiotic gene clusters. Antoine Leeuwenhoek.

[218] Ward M.G. (2016). The Regulatory Landscape for Biological Control Agents. EPPO Bull.

[219] Santos V.B., Araujo S.F., Leite L.F., Nunes L.A., Melo J.W. (2012). Soil microbial biomass and organic matter fractions during transition from conventional to organic farming systems. Geoderma.

[220] Youssef M.M.A., Eissa M.F.M. (2014). Biofertilizers and their role in management of plant parasitic nematodes. A review. E3 J. Biotechnol. Pharm. Res..

[221] Nina K., Thomas W.K., Prem S.B. (2014). Beneficial Organisms for Nutrient Uptake.

[222] Sahoo R.K., Ansari M.W., Dangar T.K., Mohanty S., Tuteja N. (2014). Phenotypic and molecular characterisation of efficient nitrogen-fixing *Azotobacter* strains from rice fields for crop improvement. Protoplasma.

[223] Singh J.S., Pandey V.C., Singh D.P. (2011). Efficient soil microorganisms: A new dimension for sustainable agriculture and environmental development. Agric. Ecosyst. Environ..

[224] Bhardwaj D., Ansari M.W., Sahoo R.K., Tuteja N. (2014). Biofertilizers function as key player in sustainable agriculture by improving soil fertility, plant tolerance and crop productivity. Microb. Cell Fact..

[225] Bashan Y., de-Bashan L.E., Prabhu S.R., Juan-Pablo H. (2014). Advances in plant growth promoting bacterial inoculant technology: Formulations and practical perspectives (1998–2013). Plant Soil.

[226] Bashan Y. (1998). Inoculants of plant growth-promoting bacteria for use in agriculture. Biotechnol. Adv..

[227] Catroux G., Hartmann A., Revellin C. (2001). Trends in rhizobial inoculant production and use. Plant Soil.

[228] Suprapta D.N. (2012). Potential of microbial antagonists as biocontrol agents against plant fungal pathogens. J. ISSAAS.

[229] Ou S.H. (1980). A look at worldwide rice blast disease control. Plant Dis..

[230] Vasudevan P., Kavitha S., Priyadarisini V.B., Babujee L., Gnanamanickam S.S., Gnanamanickam S.S. (2002). Biological control of rice diseases. Biological Control of Crop Diseases.

[231] Dubey R.C., Dubey R.C. (1993). Biopesticides: Biological control of plant pathogens, pests and weeds. A Textbook of Biotechnology.

[232] Yang J.H., Liu H.X., Zhu G.M., Pan Y.L., Xu L.P., Guo J.H. (2008). Diversity analysis of antagonists from rice-associated bacteria and their application in biocontrol of rice diseases. J. Appl. Microbiol..

[233] Law J.W.-F., Ser H.-L., Khan T.M., Chuah L.-H., Pusparajah P., Chan K.-G., Goh B.-H., Lee L.-H. (2017). The Potential of *Streptomyces* as Biocontrol Agents against the Rice Blast Fungus, *Magnaporthe oryzae* (*Pyricularia oryzae*). Front. Microbiol..

[234] Bibb M.J. (2013). Understanding and manipulating antibiotic production in actinomycetes. Biochem. Soc. Trans..

